# Repurposing BCL2 inhibitors: Venetoclax protects against acinar cell necrosis in acute pancreatitis by promoting apoptosis

**DOI:** 10.1038/s41419-025-07881-w

**Published:** 2025-07-27

**Authors:** Jacek J. Litewka, Mateusz D. Szopa, Katarzyna Fryt, Monika A. Jakubowska, Urszula Jankowska, Bozena Skupien-Rabian, Karolina Hajduk, Ewa Werner, Kinga B. Stopa, Agnieszka A. Kusiak, Daria Krzysztofik, Zbigniew Madeja, Pawel E. Ferdek

**Affiliations:** 1 Department of Cell Biology, Faculty of Biochemistry, Biophysics and Biotechnology, Jagiellonian University, ul. Gronostajowa 7, 30-387 Krakow, Poland; 2 Doctoral School of Exact and Biological Sciences, Jagiellonian University, ul. Łojasiewicza 11, 30-348 Krakow, Poland; 3 Malopolska Centre of Biotechnology, Jagiellonian University, ul. Gronostajowa 7A, 30-387 Krakow, Poland; 4 Proteomics Core Facility, Faculty of Biochemistry, Biophysics and Biotechnology, Jagiellonian University, ul. Gronostajowa 7, 30-387 Krakow, Poland; 5 Animal Facility, Faculty of Biochemistry, Biophysics and Biotechnology, Jagiellonian University, ul. Gronostajowa 7, Krakow, 30-387 Poland

**Keywords:** Cell death, Mechanisms of disease, Pancreatitis

## Abstract

Acute pancreatitis (AP), a severe inflammatory disorder of the pancreas, lacks effective pharmacological treatment. The disease is primarily driven by necrosis of pancreatic acinar cells (PACs), which intensifies inflammation and organ injury. This study explores the potential of BCL2 inhibitors, specifically Navitoclax and Venetoclax, to shift cell death pathways from necrosis to apoptosis and thereby mitigate disease severity. Ex vivo models using cerulein or ethanol/palmitoleic acid (EtOH/POA) showed that both inhibitors significantly reduced necrosis, increased apoptosis, and improved PAC viability and ATP levels. In mouse models of AP, both drugs promoted apoptosis and decreased tissue necrosis, with Venetoclax showing superior efficacy and safety. Venetoclax markedly reduced disease severity in two AP models without affecting healthy tissue or inducing thrombocytopenia. In contrast, Navitoclax caused apoptosis even in healthy tissue and triggered thrombocytopenia. Additionally, both drugs attenuated pathological Ca^2+^ responses in PACs and upregulated the expression of Ca²⁺-binding proteins S100A8/A9 and the chemokine CCL8. The latter may mediate enhanced apoptotic clearance and limit secondary necrosis, supporting the therapeutic shift from necrosis to apoptosis. Proteomic analyses revealed extensive drug-induced remodeling. In the short-term AP model, both inhibitors altered expression of proteins linked to intracellular compartments and extracellular signaling, reflecting cellular adaptation. In CP, Navitoclax upregulated ECM and lysosomal proteins while downregulating ribosomal components—indicating intensified fibrosis and suppressed protein synthesis. Venetoclax had milder effects and did not worsen fibrosis. Despite Navitoclax’s efficacy toward activated pancreatic stellate cells in vitro, it exacerbated fibrosis and tissue atrophy in CP in vivo, likely due to ongoing parenchymal damage and stellate cell activation. Together, these findings demonstrate that selective BCL2 inhibition with Venetoclax promotes apoptosis, reduces necrosis, and improves outcomes in AP, supporting its repurposing as a therapeutic strategy. However, BCL2 inhibition does not benefit CP and may aggravate fibrosis, underscoring the need for context-specific approaches.

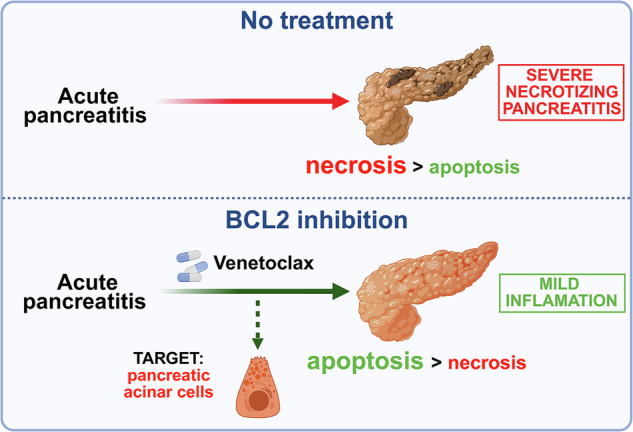

## Introduction

Acute pancreatitis (AP) is an inflammatory condition of the pancreas, most commonly triggered by alcohol abuse or gallstones, and remains a leading cause of gastrointestinal hospitalization in the United States, with an annual economic burden exceeding $9.3 billion [1]. Despite being recognized for nearly 400 years, effective treatments are limited. AP severity varies widely, from self-limiting cases to severe forms causing systemic inflammation, multi-organ failure, and mortality rates of 30–50% [2–4].

The disease is driven by necrotic death of pancreatic acinar cells (PACs), which under pathological conditions prematurely activate digestive enzymes, leading to tissue autodigestion. In contrast, apoptosis minimizes enzyme release and inflammation by preventing cellular rupture. Increased apoptosis is associated with milder AP, suggesting therapeutic potential in promoting apoptotic pathways [5, 6].

Apoptosis is regulated by BCL2 family proteins, which include anti-apoptotic (BCL2, BCL-xL, MCL1), pro-apoptotic (BAX, BAK), and BH3-only members [5]. Anti-apoptotic proteins inhibit cell death, while BAX and BAK mediate mitochondrial membrane permeabilization and caspase activation. BH3-only proteins promote apoptosis by antagonizing anti-apoptotic members.

**Fig. 1:**
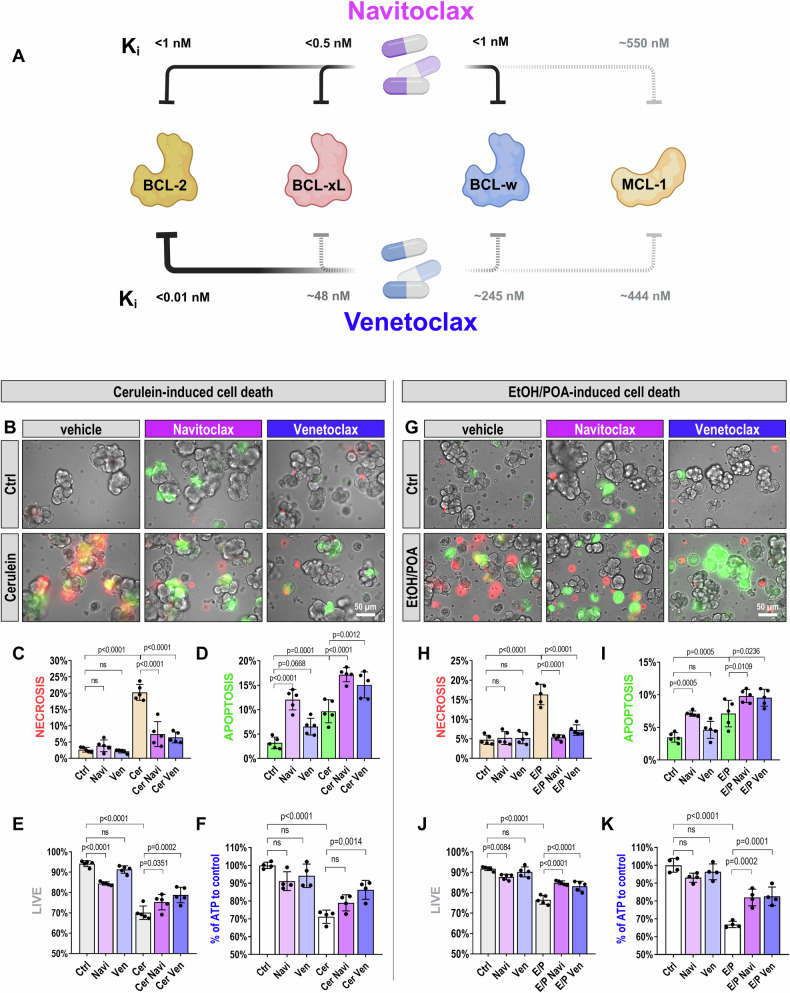
Modulation of cell death pathways by BCL2 inhibitors in PACs treated with cerulein or EtOH/POA. **A** Schematic illustration depicting the selectivity and affinity of BCL2 inhibitors, Navitoclax and Venetoclax, toward anti-apoptotic BCL2 family proteins (BCL2, BCL-xL, BCL-w, and MCL-1) [8]. Created in BioRender. **B** Representative images of mouse PACs loaded with NucView 488 and RedDot 2, pre-incubated with Navitoclax or Venetoclax at 10 μM (or DMSO control) for 15 min and then treated with cerulein at 10 nM for 1 hour. Green fluorescence of NucView 488 indicates active caspase 3 and 7, signifying apoptosis; red fluorescence of RedDot 2 indicates necrosis due to damaged cell membranes. Images taken with a 40× magnification objective. **C**−**E** Quantitative results from mouse PAC cell death assessment following pre-stimulation with Navitoclax, Venetoclax (10 μM), or control (DMSO) for 15 min, and subsequent cerulein treatment (10 nM) for 1 hour, representative images are shown in (**B**). Bar chart in (**C**) shows the percentage of necrotic cells, (**D**) the percentage of apoptotic cells, and (**E**) live cells, relative to the total cell count. Data collected from 5 independent experiments using freshly isolated mouse PACs (*N* = 5), 15 images taken per condition, with total cell counts as follows: Ctrl: 5506, Navi: 5824, Ven: 5116, Cer: 3911, Cer Navi: 3879, Cer Ven: 3406. Results are presented as mean ± SD. **F** ATP measurements in mouse PACs after pre-stimulation with Navitoclax, Venetoclax (10 μM), or control (DMSO) for 15 min, followed by cerulein treatment (10 nM) for 1 hour. Performed using the CellTiter-Glo 3D Cell Viability Assay. Results are normalized to total protein concentration and expressed as a percentage relative to control (Ctrl). Data derived from 4 separate PAC isolations (*N* = 4). Results are presented as mean ± SD. **G** Representative images of mouse PACs loaded with NucView 488 and RedDot 2, pre-incubated with Navitoclax or Venetoclax at 10 μM (or DMSO control) for 15 min and then treated with EtOH 200 mM + POA 200 μM for 1 hour. Green fluorescence of NucView 488 indicates active caspase 3 and 7, signifying apoptosis; red fluorescence of RedDot 2 indicates necrosis due to damaged cell membranes. Images taken with a 40× magnification objective. **H−J** Quantitative results from mouse PAC cell death assessment following pre-stimulation with Navitoclax, Venetoclax (10 μM), or control (DMSO) for 15 min, and subsequent treatment with EtOH 200 mM + POA 200 μM for 1 hour, representative images are shown in (**G**). Bar chart in (**H**) shows the percentage of necrotic cells, (**I**) the percentage of apoptotic cells, and (**J**) live cells, relative to the total cell count. Data collected from 5 independent experiments using freshly isolated mouse PACs (*N* = 5), 15 images taken per condition, with total cell counts as follows: Ctrl: 5682, Navi: 5616, Ven: 5858, E/P: 5316, E/P Navi: 5521, E/P Ven: 5222. Results are presented as mean ± SD. **K** ATP measurements in mouse PACs after pre-stimulation with Navitoclax, Venetoclax (10 μM), or control (DMSO) for 15 min, followed by treatment with EtOH 200 mM + POA 200 μM for 1 hour. Conducted using the CellTiter-Glo 3D Cell Viability Assay. Results are normalized to total protein concentration and expressed as a percentage relative to control (Ctrl). Data were obtained from 4 separate PAC isolations (*N* = 4). Results are presented as mean ± SD. Statistical analyses: Data distribution was assessed for normality using the Shapiro-Wilk test, confirming that the data were normally distributed. Statistical analyses were performed using one-way ANOVA followed by Sidak’s multiple comparisons test to assess differences between groups. Figure abbreviation legend: Ctrl (NaHEPES), Navi (Navitoclax 10 μM), Ven (Venetoclax 10 μM), Cer (cerulein 10 nM), Cer Navi (cerulein 10 nM + Navitoclax 10 μM), Cer Ven (cerulein 10 nM + Venetoclax 10 μM), E/P (EtOH 200 mM + POA 200 μM), E/P Navi (EtOH 200 mM + POA 200 μM + Navitoclax 10 μM), E/P Ven (EtOH 200 mM + POA 200 μM + Venetoclax 10 μM).

BCL2 inhibitors such as Venetoclax (ABT-199) and Navitoclax (ABT-263) modulate these pathways [6]. Venetoclax, FDA-approved for hematologic malignancies [7, 8], selectively targets BCL2, while Navitoclax inhibits BCL2, BCL-xL, and BCL-w (Fig. 1A) [8]. By disabling key anti-apoptotic defenses, these drugs facilitate controlled apoptosis, potentially limiting inflammation and tissue injury in AP.

Here, we explore whether BCL2 inhibition can reduce AP severity by redirecting cell death from necrosis to apoptosis—a proof-of-principle approach for therapeutic intervention.

## Results

### Navitoclax and Venetoclax modulate cell death in PACs treated with cerulein

First, we evaluated the effects of Navitoclax and Venetoclax on cell death pathways in mouse PACs using ex vivo models. One model involved stimulation with cerulein, while the other used a combination of ethanol (EtOH) and palmitoleic acid (POA) to mimic the development of acute pancreatitis induced by alcohol abuse.

Cerulein (Cer), a cholecystokinin (CCK) analog, is commonly used in AP models [9]. In our experiments, PACs were treated with 10 nM cerulein, with or without 10 µM Navitoclax or Venetoclax (Fig. 1B–F). Cerulein alone increased necrotic cell death, whereas both inhibitors significantly reduced necrosis (Fig. 1B, quantification in Fig. 1C). They also increased apoptosis (Fig. 1D), and improved overall cell viability (Fig. 1E), indicating that promoting apoptosis enhances survival in stressed PAC populations.

While both drugs significantly reduced necrosis, Navitoclax alone caused an increase in apoptosis in PACs compared to the untreated control group (Fig. 1D), accompanied by a slight decrease in the total number of live cells (Fig. 1E). In contrast, Venetoclax alone did not markedly affect cell death in the untreated cells (Fig. 1C–E).

Cell viability correlated with changes in intracellular ATP levels. Cerulein treatment alone reduced ATP levels by approximately 30% compared to the control (Fig. 1F). In contrast, treatment with Venetoclax, but not Navitoclax, significantly reversed this effect, restoring ATP levels toward those of the control group (Fig. 1F).

### Navitoclax and Venetoclax promote apoptosis in EtOH/POA-treated PACs

Similarly to cerulein, treatment with 200 mM ethanol and 200 µM palmitoleic acid (EtOH/POA), inducers of pancreatic pathology [10, 11], markedly increased necrosis in PACs (Fig. 1G, H). Co-administration of Navitoclax or Venetoclax with EtOH/POA dramatically reduced necrosis, bringing its levels in line with those of the control group. Simultaneously, there was a notable surge in apoptosis in the presence of the BCL2 inhibitors (Fig. 1I). Unlike Venetoclax, Navitoclax also significantly increased apoptosis in untreated PACs (Fig. 1I), which was associated with reduced total live cell count (Fig. 1J).

EtOH/POA treatment alone decreased intracellular ATP levels. However, both Venetoclax and Navitoclax countered this drop, boosting ATP levels toward control values (Fig. 1K).

This parallel examination of two distinct treatments in our ex vivo models suggests a consistent modulation of cell death pathways by Navitoclax and Venetoclax, providing a strong rationale for their potential application in vivo as therapeutic strategies against AP.

### Neither Venotoclax nor Navitoclax markedly disrupts Ca^2+^ homeostasis in PACs

Pathological calcium (Ca^2+^) responses play a critical role in the development of AP. In PACs, elevated cytosolic Ca^2+^ leads to premature activation of digestive enzymes, which can degrade cellular components and result in necrosis and autodigestion of pancreatic tissue [12, 13]. This typically occurs due to excessive Ca^2+^ influx, uncontrolled release from intracellular stores, or impaired sequestration back into stores, disrupting cellular balance and triggering damaging cascades that exacerbate pancreatitis severity and promote systemic inflammation [13].

**Fig. 2:**
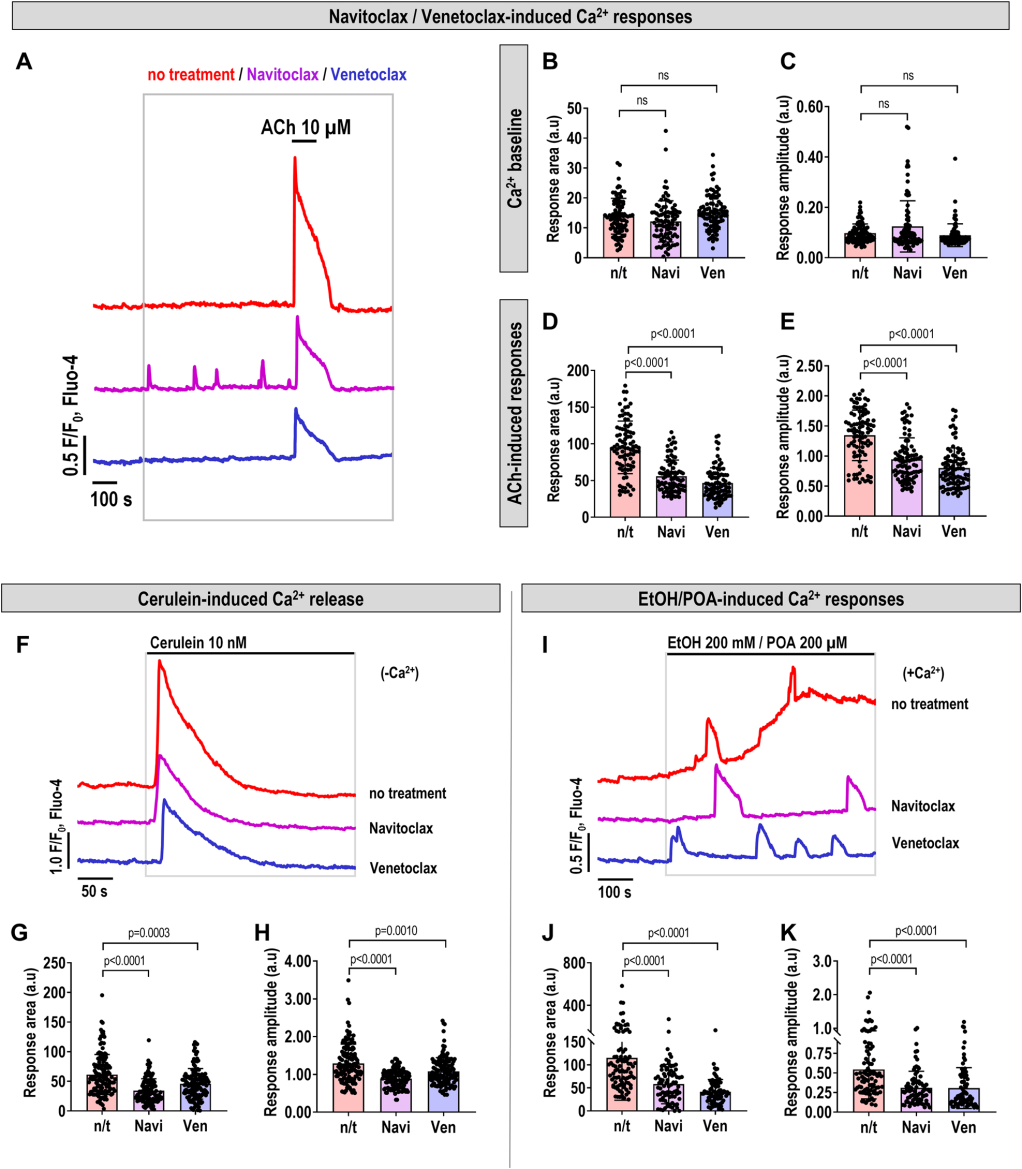
Calcium signaling in PACs treated with BCL2 inhibitors. **A** Representative traces of intracellular Ca^2+^ responses recorded in mouse PACs upon treatment with Navitoclax, Venetoclax (10 μM), or no treatment (control) for 10 min (between 200−800 seconds of the recording), followed by application of high concentrations of acetylcholine (ACh 10 μM) for 100 seconds (between 800−900 seconds of the experiment). Data for the representative traces and subsequent analyses were obtained from PACs loaded with Fluo-4 AM dye, in a continuous perfusion flow system using NaHEPES buffer, from six independent experiments for each group (*N* = 6, *n* = 90 cells). For Navitoclax and Venetoclax conditions, cells were derived from 3 independent isolations of PACs, with each isolation contributing to 2 experiments, 15 cells per experiment. In contrast, cells used for control experiments were obtained from a single isolation, with all 6 experiments conducted on this sample. **B** Normalized area under the Ca^2+^ signaling traces from 200−800 seconds in PACs after acute treatment with Navitoclax, Venetoclax (10 μM), or no treatment (control); representative traces are depicted in (**A**). Results are presented as mean ± SD. **C** Normalized maximal amplitude from Ca^2+^ signaling traces between 200−800 seconds in PACs following acute application of Navitoclax, Venetoclax (10 μM), or no treatment (control); representative traces are depicted in (**A**). Results are presented as mean ± SD. **D** Normalized area under the Ca²⁺ signaling traces from 800−900 seconds in PACs during application of high concentration of ACh (10 µM) after pretreatment with Navitoclax, Venetoclax (10 µM), or no pretreatment (control); representative traces are depicted in (**A**). Results are presented as mean ± SD. **E** Normalized maximal amplitude from Ca²⁺ signaling traces between 800-900 seconds in PACs during application of high concentration of ACh (10 µM) after pretreatment with Navitoclax, Venetoclax (10 µM), or no pretreatment (control); representative traces are depicted in (**A**). Results are presented as mean ± SD. **F** Representative traces of intracellular Ca^2+^ responses recorded in mouse PACs treated with cerulein (10 nM) for 5 min (between 100−400 seconds of the recording); cells were preincubated for 15 min with Navitoclax, Venetoclax, or without treatment (non-treated, n/t) prior to the experiment. The BCL2 inhibitors remained present throughout the entire duration. Data for the representative traces and subsequent analyses were obtained from PACs stained with Fluo-4 AM dye, in a continuous perfusion flow system using NaHEPES buffer devoid of Ca^2+^ ions, from 4 independent experiments, each from a different cell isolation (*N* = 4, Navitoclax *n* = 120, Venetoclax *n* = 135, n/t *n* = 135 cells). **G** Normalized area under the Ca²⁺ signaling traces from 100−400 seconds in PACs treated with cerulein (10 nM) for 5 min after pretreatment with BCL2 inhibitors; representative traces are depicted in (**F**). Results are presented as mean ± SD. **H** Normalized maximal amplitude from Ca^2+^ signaling traces between 100−400 seconds in PACs treated with cerulein (10 nM) for 5 min after pretreatment with BCL2 inhibitors; representative traces are depicted in F. Results are presented as mean ± SD. **I** Representative traces of intracellular Ca^2+^ responses recorded in mouse PACs treated with EtOH 200 mM + POA 200 μM for 10 min (between 200−800 seconds of the recording); cells were preincubated for 15 min with Navitoclax, Venetoclax, or without treatment (non-treated, n/t) prior to the experiment. The BCL2 inhibitors remained present throughout the entire duration. Data for the representative traces and subsequent analyses were obtained from PACs stained with Fluo-4 AM dye, in a continuous perfusion flow system using NaHEPES buffer with 1 mM Ca^2+^, from 3 independent experiments, each from a different cell isolation (*N* = 3, Navitoclax *n* = 80, Venetoclax *n* = 80, n/t *n* = 90 cells). **J** Normalized area under the Ca²⁺ signaling traces from 200-800 seconds in PACs treated with EtOH 200 mM + POA 200 μM for 10 min after pretreatment with BCL2 inhibitors; representative traces are depicted in (**I**). Results are presented as mean ± SD. **K** Normalized maximal amplitude from Ca^2+^ signaling traces between 200−800 seconds in PACs treated EtOH 200 mM + POA 200 μM for 10 min after pretreatment with BCL2 inhibitors; representative traces are depicted in (**I**). Results are presented as mean ± SD. Statistical analyses: Data distribution was assessed for normality using the Shapiro-Wilk test, which confirmed that the data were not normally distributed. Consequently, non-parametric statistical analyses were performed using the Kruskal-Wallis test followed by Dunn’s post hoc test to assess differences between groups. Figure abbreviation legend: n/t (non-treatment, that is in NaHEPES), Navi (Navitoclax 10 μM), Ven (Venetoclax 10 μM), Cer (cerulein 10 nM), ACh 10 μM (acetylcholine 10 μM), -Ca^2+^ (NaHEPES without Ca^2+^ ions).

Given the above, here we explore how Navitoclax and Venetoclax affect intracellular Ca^2+^ homeostasis in PACs, which is known to be influenced by BCL2 proteins or their inhibition [14–17]. While Venetoclax showed no notable Ca^2+^ changes (Fig. 2A), Navitoclax triggered only sporadic Ca^2+^ transients that did not significantly affect average response amplitudes or areas (Fig. 2B, C). This subtle effect of Navitoclax is likely linked to its inhibition of BCL-xL [18].

Using acetylcholine (ACh, 10 µM) as a control to induce Ca^2+^ release from intracellular stores, we noted that both BCL2 inhibitors modestly reduced ACh-induced Ca^2+^ responses, seen as a reduction in the response area (Fig. 2D) and amplitude (Fig. 2E). These results indicate that while neither drug directly causes significant Ca^2+^ release, they can modulate how cells respond to other stimuli that typically increase intracellular Ca^2+^ levels.

### Venetoclax and Navitoclax reduce Ca^2+^ responses in PACs induced by cerulein and EtOH/POA

We further explored how Navitoclax and Venetoclax affect pathophysiological Ca^2+^ responses in PACs triggered by cerulein and EtOH/POA. Under Ca^2+^-free extracellular conditions—with active extrusion mechanisms maintained—control cells (red trace) showed marked cytosolic Ca^2+^ elevations due to CCK receptor overstimulation and subsequent ER release via ryanodine receptor (RyR) and inositol triphosphate receptor (IP3R) (Fig. 2F). In contrast, both Navitoclax (violet) and Venetoclax (blue) substantially reduced these responses. Quantitative analyses of response area (Fig. 2G) and peak amplitude (Fig. 2H) confirmed significant suppression of Ca^2+^ signals, demonstrating effective modulation of intracellular Ca^2+^ dynamics by both inhibitors.

EtOH/POA treatment, in the presence of extracellular Ca^2+^, resulted in a sustained Ca^2+^ plateau (Fig. 2I), dependent on the entry of Ca^2+^ from the extracellular environment [19]. Navitoclax or Venetoclax significantly lowered this plateau, as evidenced by the representative traces (Fig. 2I) and quantified data for average response area and amplitude (Fig. 2J, K). The ability to attenuate both immediate and prolonged Ca^2+^ elevations highlights the potential of BCL2 inhibitors to regulate not just transient, but also chronic pathological Ca^2+^ elevations in PACs. These findings indicate that beyond their immediate role in apoptosis regulation, these drugs may impact broader cellular stability and function in the context of pancreatic diseases.

### Venetoclax decreases the severity of AP in the cerulein- and EtOH/POA-induced mouse models

Next, we utilized a cerulein-induced mouse model to validate the effects of Navitoclax and Venetoclax on AP in vivo. This model, which closely mimics the clinical manifestations of human AP [9], involved repeated cerulein injections to induce pancreatitis, with some mice given Navitoclax, Venetoclax, or vehicle control.

**Fig. 3:**
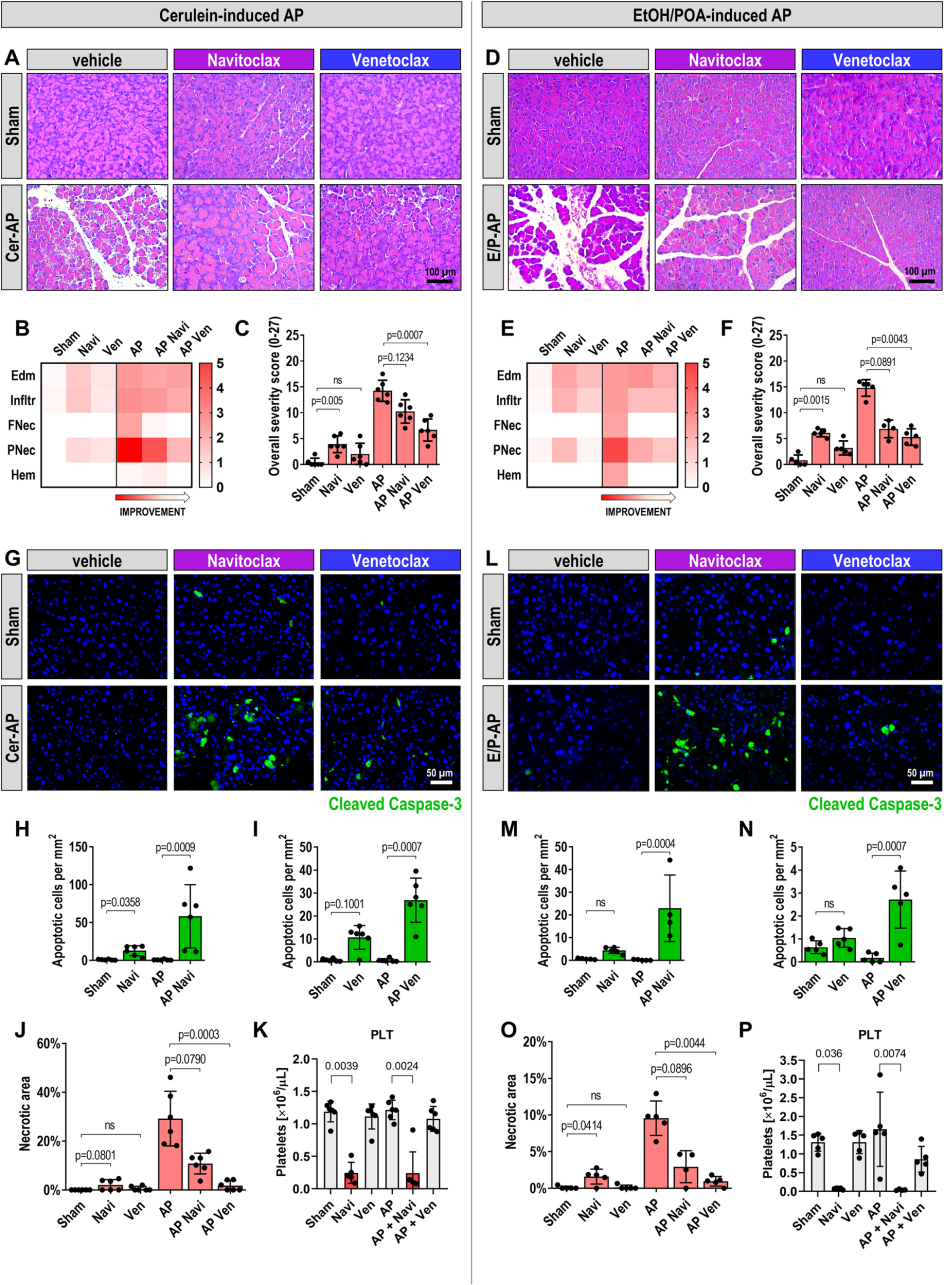
Navitoclax and Venetoclax in cerulein- and EtOH/POA-induced models of AP. **A** Representative hematoxylin and eosin (H&E) stained images of mouse pancreata from the cerulein-induced AP model. Both Navitoclax and Venetoclax significantly reduced tissue injury and edema. Scale bar: 100 µm. Images taken with a 20 × magnification objective. **B** Heatmap displaying the histological evaluation scores of pancreata from the cerulein-induced AP model. Evaluated parameters included edema (Edm, 0/1/2/3), inflammatory infiltration (Infltr, 0/1/2/3), fat necrosis (FNec, 0/3/5/7), parenchymal necrosis (PNec, 0/3/5/7), and hemorrhages (Hem, 0/3/5/7) [20]. **C** Overall histological severity score (0−27) for each group in the cerulein-induced AP model, derived from the average assessments of two blinded investigators. Results are presented as mean ± SD. **D** Representative H&E stained images of mouse pancreata from the EtOH/POA-induced AP model. Application of Navitoclax and Venetoclax resulted in markedly reduced tissue injury and edema. Scale bar: 100 µm. **E** Heatmap depicting the histological evaluation scores of pancreata from the EtOH/POA-induced AP model. Scores for edema (Edm, 0/1/2/3), inflammatory infiltration (Infltr, 0/1/2/3), fat necrosis (FNec, 0/3/5/7), parenchymal necrosis (PNec, 0/3/5/7), and hemorrhages (Hem, 0/3/5/7) are presented, similar to B [20]. **F** Overall histological severity score (0−27) for each group in the EtOH/POA-induced AP model, calculated from blinded assessments by two independent investigators. Results are presented as mean ± SD. **G** Representative immunohistochemical staining images of cleaved caspase-3 in pancreata from the cerulein-induced AP model, indicating apoptosis. Scale bar: 50 µm. **H**-**I** Quantification of apoptotic cells per mm^2^ in the cerulein-induced AP model, determined through immunohistochemical staining for cleaved caspase-3. Analyses were performed on whole tissue sections and results are presented as mean ± SD. The graphs show the same values for Sham and AP. **J** Percentage of necrotic areas in mouse pancreata from the cerulein AP model. Results are presented as mean ± SD. **K** Platelet counts from postmortem blood samples of mice from the cerulein AP model, analyzed using the ABC VET hematology analyzer. Results presented as mean ± SD. **L** Representative images of immunohistochemical staining of cleaved caspase-3 in pancreata from the EtOH/POA-induced AP model, indicating apoptosis. Scale bar: 50 µm. Images captured with a 40 × magnification objective. **M**, **N** Quantification of apoptotic cells per mm^2^ in the EtOH/POA-induced AP model, analyzed on whole tissue sections and results presented as mean ± SD. The graphs show the same values for Sham and AP. **O** Percentage of necrotic areas in mouse pancreata from the EtOH/POA-induced AP model. Results presented as mean ± SD. **P** Platelet counts from postmortem blood samples of mice from the EtOH/POA-induced AP model, analyzed using the ABC VET hematology analyzer. Results presented as mean ± SD. Statistical analyses: For the cerulein-induced AP model, *n* = 6 for all groups; for the EtOH/POA-induced AP model, *n* = 5, except for the AP Navi group with *n* = 4. The statistical analyses were performed using the non-parametric Kruskal-Wallis test followed by Dunn’s post hoc test, applied due to some datasets not being normally distributed or when *n* < 6. Figure Abbreviation Legend: Sham (saline + vehicle), Navi (saline + Navitoclax), Ven (saline + Venetoclax), AP (cerulein or EtOH/POA + vehicle), AP + Navi (cerulein or EtOH/POA + Navitoclax), AP + Ven (cerulein or EtOH/POA + Venetoclax).

Histological assessments revealed typical AP characteristics (Fig. 3A). Edema, immune infiltration, fat necrosis, parenchymal necrosis, and hemorrhages were scored, visualized in a heatmap (Fig. 3B), and summarized as the overall severity score (Fig. 3C) [20]. Both Navitoclax and Venetoclax improved these parameters, especially parenchymal necrosis, with significant reductions in the overall severity score noted for Venetoclax (Fig. 3C). While Venetoclax did not worsen the severity score in healthy tissues, Navitoclax slightly increased the score for edema and infiltration, suggesting some adverse effects (Fig. 3B, C).

To test the reproducibility of these findings under different pathophysiological conditions, we applied a second animal model of AP induced by EtOH and POA [21]. Histological evaluations (Fig. 3D) and scoring of AP indicators (Fig. 3E) showed consistent improvements with both treatments. Particularly, Venetoclax significantly lowered the overall severity score compared to the untreated group (Fig. 3F), while Navitoclax demonstrated a notable reduction in the severity score that approached but did not reach statistical significance (Fig. 3F). Additionally, Navitoclax slightly increased the severity score in healthy pancreatic tissue (Fig. 3F).

AP is associated with systemic inflammation and secondary lung injury, as reflected by increased inflammatory cell infiltration and alveolar damage (Supplementary Figs. 1A and 1C) [22]. While treatment with Navitoclax or Venetoclax alone occasionally increased lung injury scores under baseline conditions, administration of these inhibitors during AP, particularly Venetoclax, significantly reduced lung injury scores in both experimental models (Supplementary Figs. 1B and 1D).

### Venetoclax increases apoptosis and reduces necrosis in the cerulein- and EtOH/POA-induced mouse models of AP

Our study further explored how Navitoclax and Venetoclax influence cell death pathways in vivo. In the cerulein-induced model of AP, immunohistochemical staining for cleaved caspase-3 showed minimal apoptosis in the AP-only group, yet treatment with either Navitoclax or Venetoclax significantly enhanced apoptotic markers in pancreatic tissues (Fig. 3G). Quantitative analyses revealed a marked increase in apoptotic cells per mm^2^ in Navitoclax or Venetoclax treated groups compared to untreated AP (Fig. 3H–I), alongside notable reductions in necrotic tissue, particularly with Venetoclax (Fig. 3J). Navitoclax showed only a trend toward reducing necrosis (Fig. 3J).

Consistent with previous findings of thrombocytopenia resulting from BCL-xL inhibition by Navitoclax [23, 24], we observed significant platelet reductions in all Navitoclax-treated animals, a side effect not seen with Venetoclax (Fig. 3K).

In the EtOH/POA-induced AP model, Navitoclax and Venetoclax similarly increased apoptosis and decreased necrosis (Fig. 3L–O). Immunohistochemical analysis confirmed an increase in apoptotic cells following treatment (Fig. 3L–N), with a corresponding decrease in necrotic areas in the AP Ven group (Fig. 3O). Additionally, platelet analysis confirmed that thrombocytopenia occurred exclusively in the animals treated with Navitoclax (Fig. 3P).

### Pharmacological BCL2 inhibition affects pancreatic stellate cells

**Fig. 4:**
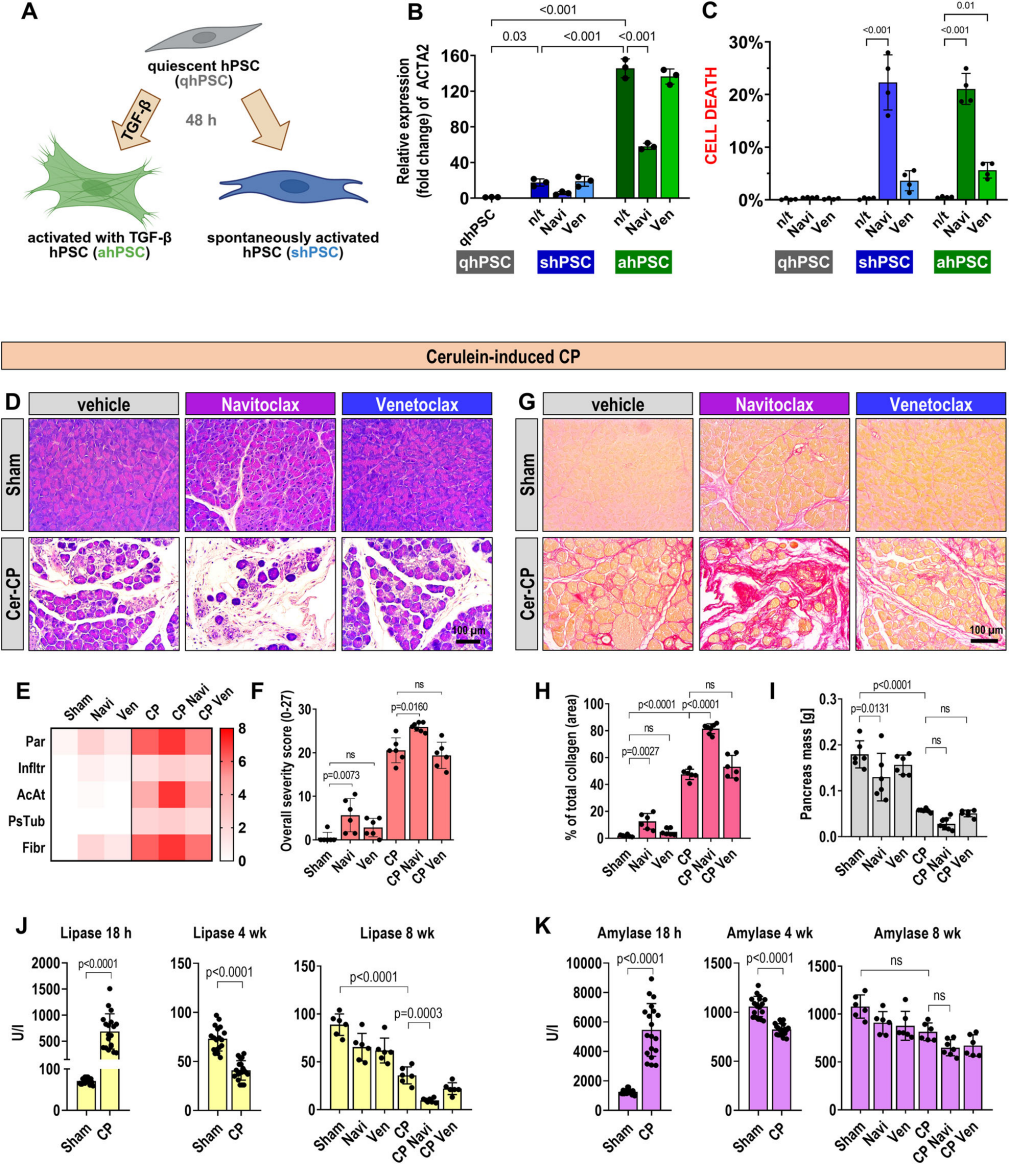
Navitoclax and Venetoclax in the cerulein-induced model of CP. **A** Schematic representation of the human pancreatic stellate cell (hPSC) activation model. Created with BioRender. **B** Relative *ACTA2* mRNA expression in hPSCs at different activation stages and following treatment with 100 nM Navitoclax or Venetoclax. Data are presented as fold change relative to quiescent hPSCs (qhPSCs) (*N* = 3). Values represent mean ± SD. Statistical significance was determined using one-way ANOVA with Sidak’s post hoc test. **C** Cell death in hPSCs with different activation states after 48 hour treatment with 100 nM Navitoclax or Venetoclax, shown as the percentage of dead cells relative to total cell number. Data from 4 independent experiments (*N* = 4); 8 images per condition. Total cell counts: qhPSC: 3335, qhPSC + Ven: 3705, qhPSC + Navi: 3292, shPSC: 1140, shPSC + Ven: 1117, shPSC + Navi: 1146, ahPSC: 1241, ahPSC + Ven: 1107, ahPSC + Navi: 1141. Results are shown as mean ± SD. Statistical analysis performed using one-way ANOVA with Sidak’s post hoc test. **D** Representative images of hematoxylin and eosin (H&E) stained mouse pancreata from the cerulein-induced CP model. Tissue injury and atrophy are particularly evident in the groups with induced CP, especially with additional administration of Navitoclax. Scale bar: 100 µm. **E** Heatmap depicting the individual parameters included in the histological evaluation of tissues from the CP model, based on H&E staining. Legend: Parenchymal integrity (Par, 0/3/5/7), inflammatory infiltration (Infltr, 0/1/2/3), acinar atrophy (AcAt, 0/3/5/7), pseudotubular complexes (PsTub, 0/1/2/3), and fibrosis (Fibr, 0/3/5/7). Adapted from previous publications and modified [20, 25]. **F** Overall histological severity score (0−27) for each group from the CP model, based on H&E staining. Results presented as mean ± SD. Statistical analysis was performed using the non-parametric Kruskal-Wallis test followed by Dunn’s post hoc test. **G** Representative images of Sirius Red staining of mouse pancreata from the cerulein-induced CP model, which highlights total collagen in the tissue. Increased fibrosis is notable across all groups with induced CP, particularly significant upon additional administration of Navitoclax. Scale bar: 100 µm. **H** Percentage of tissue area from the CP model represented by total collagen, calculated across entire tissue scans from Sirius Red-stained sections. Results are presented as mean ± SD. Statistical analysis was performed using one-way ANOVA followed by Sidak’s post hoc test. **I** Mass of pancreata from the CP model, weighed at postmortem dissection. Results are presented as mean ± SD. Statistical analysis was performed using the non-parametric Kruskal-Wallis test followed by Dunn’s post hoc test. **J**, **K** Enzymatic activity measurements of lipase (**G**) and amylase (**H**) from mouse plasma in the CP model. Measurements at 18 h post the first cerulein administration (18 h), after the 4th week (4 wk), and postmortem at the end of the 8th week (8 wk). Results are presented as mean ± SD. Statistical analyses for lipase at 8 weeks were performed using one-way ANOVA followed by Sidak’s post hoc test, and for amylase at 8 weeks, the Kruskal-Wallis test followed by Dunn’s post hoc test For earlier time points, unpaired *t*-tests with Welch’s correction were applied. For the cerulein-induced CP model, *n* = 6 for all groups, except for the CP Navi group with *n* = 7. Figure Abbreviation Legend: Sham (saline + vehicle), Navi (saline + Navitoclax), Ven (saline + Venetoclax), CP (cerulein + vehicle), CP + Navi (cerulein + Navitoclax), CP + Ven (cerulein + Venetoclax).

Given the pivotal role of pancreatic stellate cells (PSCs) in promoting fibrosis during pancreatic injury, we examined the effects of BCL2 inhibitors on activation of human PSCs (hPSCs) by applying them under different conditions: to quiescent cells (qhPSCs), or simultaneously with the induction of spontaneous activation in hPSCs (shPSCs), or with TGF-β stimulation in activated hPSCs (ahPSCs) (Fig. 4A).

Navitoclax, but not Venetoclax, significantly reduced the expression of α-smooth muscle actin (α-SMA, encoded by *ACTA2*), a hallmark of PSC activation (Fig. 4B). This indicates that Navitoclax has a deactivating or cytotoxic effect specifically on activated hPSCs. Consistent with this, Navitoclax induced significant cell death in both shPSC and ahPSC populations, while having no significant effect on qhPSCs (Fig. 4C). Venetoclax showed only a relatively mild effect, limited to the ahPSC population.

Further analysis of intracellular calcium homeostasis revealed that both Navitoclax and Venetoclax modulated basal Ca^2+^ levels in qhPSCs (Supplementary Fig. 2A–C). Additionally, the diminished final ACh-induced Ca^2+^ responses observed with Navitoclax treatment may indicate partial exhaustion of intracellular calcium stores (Supplementary Fig. 2D–E). Furthermore, only Navitoclax on its own exerted a mild effect on Ca^2+^ homeostasis in ahPSCs (Supplementary Fig. 2F–J).

### Venetoclax does not improve and Navitoclax worsens fibrosis in the cerulein-induced model of CP

Since Navitoclax selectively targets activated PSCs, inducing their death and downregulating markers of activation, we proceeded to evaluate the impact of BCL2 inhibition in vivo, using a cerulein-induced model of chronic pancreatitis (CP), to assess whether this strategy could mitigate pancreatic fibrosis.

Histological analysis displayed significant pancreatic injury and atrophy in the CP groups, especially notable for treatment with Navitoclax (Fig. 4D). We assessed the following histological parameters: parenchymal integrity, inflammatory infiltration, acinar atrophy, pseudotubular complexes, and fibrosis, which were scored and presented in a heatmap (Fig. 4E) [20, 25]. These parameters contributed to the overall severity score ranging from 0 to 27 (Fig. 4F). Navitoclax increased the severity score in both the control and CP groups, whereas Venetoclax did not significantly alter the score in either healthy or inflamed tissues (Fig. 4F).

Collagen staining with Sirius Red showed increased fibrosis in CP compared to the control (Fig. 4G–H). While Venetoclax did not affect collagen deposition, Navitoclax significantly increased it in both the control and CP tissues.

Pancreatic atrophy was also evaluated by weighing the pancreata at postmortem, revealing a significant reduction in pancreatic mass in CP compared to controls (Fig. 4I). Navitoclax treatment resulted in a decrease in pancreatic mass in the control group, while Venetoclax had no effect.

We also measured lipase and amylase activity in mouse plasma at three time points (Fig. 4J, K). Both enzymes significantly increased 18 h post-induction, with notable reductions after 4 and 8 weeks between the CP and Sham groups. Notably, Navitoclax treatment further decreased lipase activity compared to CP alone at the experiment’s end.

The findings from the CP model contrast those from the AP models – while Venetoclax is effective in managing acute inflammation, it shows no significant benefit in CP; conversely, Navitoclax treatment leads to aggravated pancreatic damage.

### Proteomic changes in acute and chronic pancreatitis

**Fig. 5:**
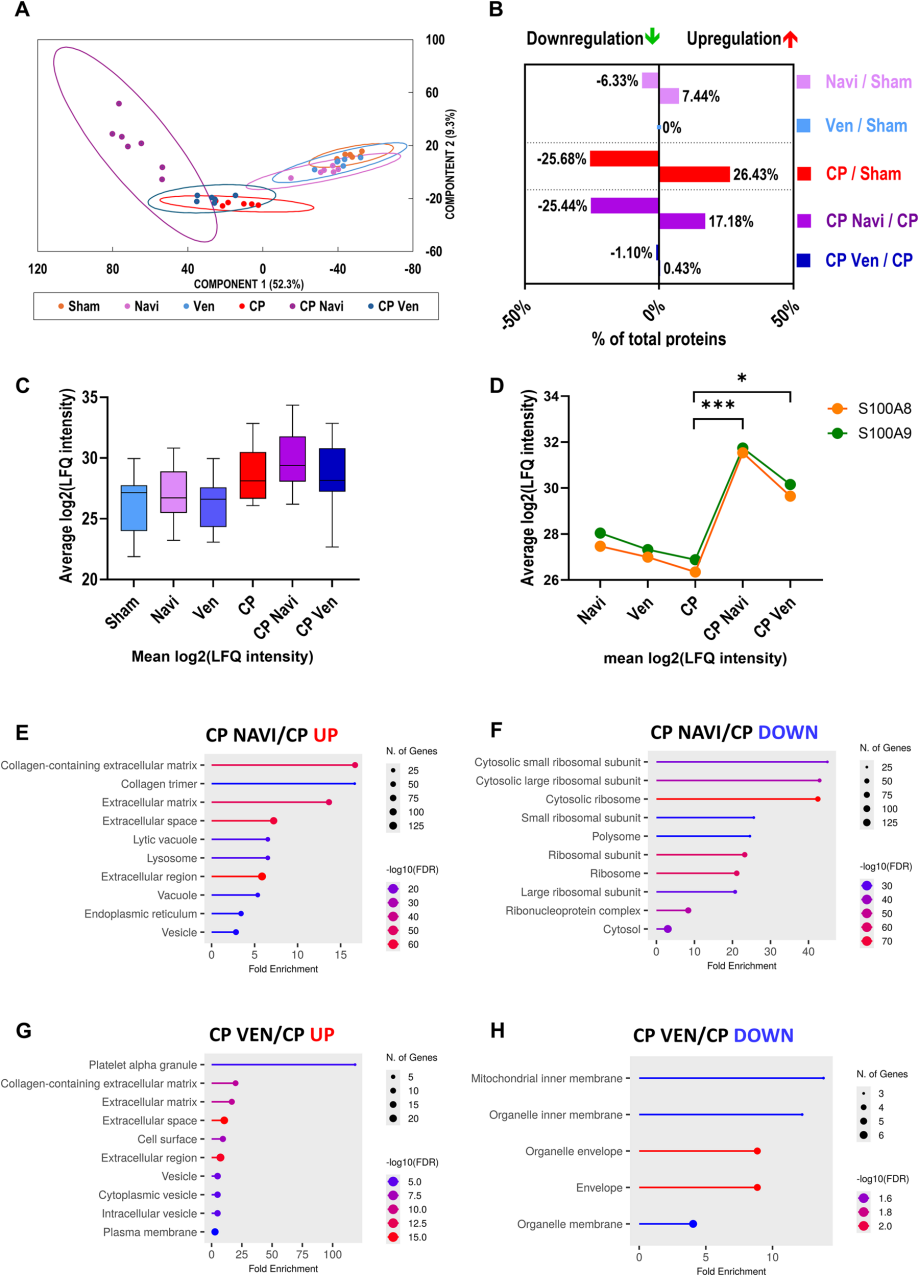
Proteomic analysis in the cerulein-induced CP model. **A** Principal component analysis (PCA) of all samples based on their proteomic profiles, displayed in various colors to differentiate experimental groups (*n* numbers as in Fig. 4: *n* = 6 for all groups, except for the CP Navi group with *n* = 7). **B** Percentage of differentially expressed proteins identified based on at least two peptides. Comparisons were conducted using Student’s *t*-test followed by permutation-based false discovery rate (FDR) correction (*q*-value < 0.05). A minimum of four valid label-free quantification (LFQ) intensity values in at least one of the two tested groups were required for each test. **C** Box plot depicting the distribution of collagen protein intensities across different experimental groups. The average log2-transformed LFQ intensity values for collagen proteins (COL1A1, COL1A2, COL3A1, COL4A1, COL4A2, COL5A1, COL5A2, COL6A1, COL6A2, COL6A5, COL6A6, COL12A1, COL14A1, COL15A1, COL18A1) were calculated and plotted within each group. **D** The average levels of proteins S100A8 and S100A9 in different experimental groups. Mean values were calculated from log2-transformed LFQ intensities. Both proteins were not quantified in the Sham group. Results are presented as mean values. Statistical analysis was performed using Student’s *t*-test. *q  < 0.05; ***q  < 0.001. Statistical significance indicated in the graph applies to both proteins analyzed. **E** Gene Ontology (GO) analysis of Cellular Component enrichment for proteins upregulated (fold change ≥ 2.0) in the CP Navi vs. CP comparison. **F** GO analysis of Cellular Component enrichment for proteins downregulated (fold change ≥ 2.0) in the CP Navi vs. CP comparison. **G** GO analysis of Cellular Component enrichment for proteins upregulated (fold change ≥ 1.2) in the CP Ven vs. CP comparison. **H** GO analysis of Cellular Component enrichment for proteins downregulated (fold change ≥ 1.2) in the CP Ven vs. CP comparison. Analyses in (**E**–**H**) were performed using ShinyGO v0.82 [62].

Proteomic analysis revealed distinct changes in AP and CP compared to Sham controls. Principal component analysis (PCA) confirmed clear clustering between groups (Supplementary Fig. 3A for AP; Fig. 5A for CP). In AP, 47.87% of proteins were upregulated and 12.92% were downregulated (Supplementary Fig. 3B), with upregulated proteins enriched in nuclear and cytosolic compartments (Supplementary Fig. 4A), and downregulated proteins related to mitochondrial structures (Supplementary Fig. 4B), indicating activation of stress responses and mitochondrial dysfunction. In CP, 26.43% of proteins were upregulated and 25.68% downregulated (Fig. 5B), with upregulated proteins associated with extracellular matrix remodeling and the actin cytoskeleton (Supplementary Fig. 4C), and downregulated proteins linked to ribosomal, mitochondrial, and ER structures (Supplementary Fig. 4D), consistent with impaired protein synthesis and fibrotic progression. Collagen expression was markedly elevated in all CP groups (Fig. 5C).

### Proteomic insights into the effects of Navitoclax and Venetoclax on the pancreas

Navitoclax induced more extensive proteomic changes than Venetoclax in both acute and chronic pancreatitis models (Fig. 5A, B; Supplementary Fig. 3A, B). In AP, treatment with either inhibitor significantly increased CCL8 expression compared to sham controls (Supplementary Fig. 3C). Both drugs also upregulated S100A8 and S100A9 proteins (Supplementary Fig. 3D). These proteins were also upregulated by both drugs in the CP model (Fig. 5D).

GO enrichment analysis showed that Navitoclax treatment acutely increased proteins associated with extracellular structures, intracellular compartments (including nuclear and organelle lumens), and protein-containing complexes (Supplementary Fig. 3E). Similarly, Venetoclax treatment enriched proteins involved in extracellular matrix organization, vesicular trafficking, and endomembrane system components (Supplementary Fig. 3F).

In the CP model, Navitoclax exerted a markedly stronger effect on protein expression compared to Venetoclax, particularly under inflammatory conditions. GO Cellular Component enrichment analysis revealed that Navitoclax treatment upregulated proteins associated with extracellular structures (collagen-containing extracellular matrix, collagen trimer, extracellular matrix, extracellular space, extracellular region), lysosomal compartments (lytic vacuole, lysosome, vacuole), the endoplasmic reticulum (ER), and vesicles (Fig. 5E). Conversely, Navitoclax downregulated proteins related to ribosomal subunits, polysomes, and ribonucleoprotein complexes, reflecting suppression of protein synthesis pathways (Fig. 5F).

Venetoclax upregulated proteins associated with ECM components, platelet alpha granules, and vesicle-related compartments, indicating enhanced tissue remodeling and secretory activity (Fig. 5G), while mitochondrial and organelle membrane proteins were downregulated, consistent with BCL-2–mediated apoptosis (Fig. 5H).

## Discussion

Our study advances the investigation of next-generation BCL2 inhibitors, Navitoclax (ABT-263) and Venetoclax (ABT-199), in preclinical models of acute pancreatitis. Both compounds showed strong ex vivo efficacy, significantly increasing apoptosis and reducing necrosis in acinar cells exposed to cerulein or EtOH/POA (Fig. 1B–E, G–J). Notably, they also dampened the excessive Ca^2+^ responses that drive acinar cell damage in AP—a key factor in the premature activation of digestive enzymes. The observed ATP increase (Fig. 1F, K) likely reflects both improved PAC survival and reduced energy demand, as decreased pathological Ca^2+^ signaling lowers the activity of ATP-dependent calcium pumps [10, 26].

The Ca^2+^-modulating effects of BCL2 inhibitors are dose-dependent. In our previous work, low Venetoclax concentrations did not significantly affect Ca^2+^ signaling in PACs [18], suggesting that higher doses – still attainable in vivo [27, 28]—are needed to alter Ca^2+^ homeostasis. BCL2 interacts with the IP3 receptor (IP3R) via its BH4 domain, limiting excessive Ca^2+^ release [29, 30]; its inhibition may increase BCL2 availability to bind IP3R and modulate Ca^2+^ flux. Anti-apoptotic BCL2 proteins also regulate RyR and SERCA channels at the ER [31, 32]. We previously showed that BCL2 loss enhances Ca^2+^ extrusion via the plasma membrane Ca^2+^-ATPase (PMCA) [15], with ABT-199 (Venetoclax) showing a similar but weaker effect [18]. This enhanced PMCA activity likely contributes to the reduced Ca^2+^ responses observed in our study (Fig. 2F–K). Thus, the protective effects of BCL2 inhibitors in AP are mediated, at least in part, by modulation of intracellular Ca^2+^ signaling – a mechanism also targeted by CRAC channel inhibitors [33–35].

Although BCL2 inhibitors targeting BCL-xL can independently trigger Ca^2+^ responses, in PACs these effects were observed only with Navitoclax and were relatively minor (Fig. 2A). While it remains unclear whether these subtle changes impact therapeutic efficacy, our findings suggest Navitoclax may not be an optimal candidate for AP treatment.

**Fig. 6:**
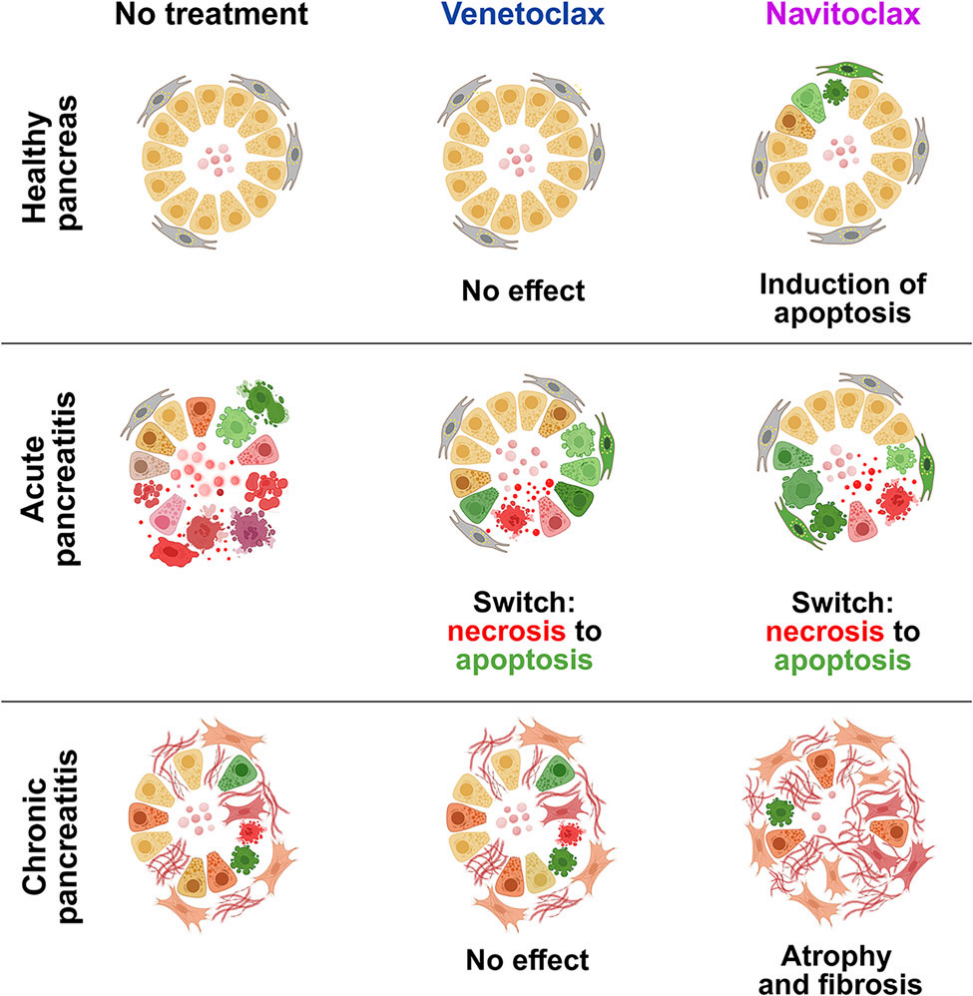
Schematic summary of the effects of BCL2 inhibition in the exocrine pancreas in healthy tissue, acute pancreatitis (AP), and chronic pancreatitis (CP). Visual representation of pancreatic tissue structure and acinar cell status under three conditions: healthy pancreas, acute pancreatitis (AP), and chronic pancreatitis (CP), in the presence or absence of BCL2 inhibitors Venetoclax and Navitoclax. Top row: In the healthy pancreas, Venetoclax has no or minimal effect, while Navitoclax induces low-level apoptosis in acinar cells (green cells), despite the absence of pathological stimuli. Middle row: In AP, which is characterized by prominent necrosis (red cells) and inflammation (left), both drugs reduce necrosis and promote apoptosis (green cells), improving tissue integrity. Venetoclax is particularly effective, shifting cell death from necrosis to apoptosis with minimal disruption to surrounding tissue. Bottom row: In CP, defined by tissue atrophy, chronic inflammation, and fibrosis (left), Venetoclax exerts limited impact on disease progression. Navitoclax, however, exacerbates acinar cell apoptosis and promotes fibrotic remodeling through stellate cell activation, leading to further parenchymal loss and architectural deterioration. Illustration created with BioRender.com.

In vivo, Venetoclax significantly increased apoptosis and reduced tissue necrosis in two AP models, effectively shifting severe inflammation to a milder phenotype (Fig. 3C and F). Its superior efficacy, along with fewer side effects—attributable to selective BCL2 inhibition without inducing thrombocytopenia or damaging healthy tissue (Fig. 3K, P and I, N)—support its preference over Navitoclax for AP therapy. To maximize efficacy during early injury, the drugs were purposely administered prior to AP induction, aligning with peak plasma levels ~6 h post-dose [27]. Although preventive, this design provides proof-of-principle that cell death modulation is achievable in AP (Fig. 6). Given that human AP typically lasts several days to weeks [36], the clinical treatment window is much wider, allowing for more controlled management of the condition in clinical settings.

Conversely, in CP, BCL2 inhibitors did not improve outcomes. Although Navitoclax showed selective cytotoxicity toward activated PSCs in vitro—suggesting potential anti-fibrotic effects—its impact in vivo was detrimental. Navitoclax increased acinar cell apoptosis, impaired regeneration, and worsened fibrosis. Promoting apoptosis in chronically damaged tissue may have limited therapeutic value, as apoptosis often predominates over necrosis in human CP [37, 38]. Additionally, Navitoclax-induced apoptosis in healthy acinar cells likely contributes to further parenchymal loss and fibrosis via stellate cell activation [39–41], a process that may override its effect observed in vitro toward activated PSCs, as tissue injury continuously drives the emergence of new PSCs.

Proteomic profiling revealed that, in the short-term AP model, Navitoclax increased the expression of proteins associated with nuclear structures, organelle lumens, the cytosol, and extracellular space, indicating broad cellular stress responses and active tissue remodeling following apoptosis induction (Supplementary Fig. 3E). Venetoclax produced a similar enrichment pattern, but more focused on, vesicular trafficking, and endomembrane systems (Supplementary Fig. 3F). This suggests activation of secretory pathways and adaptive responses with minimal nuclear remodeling, indicating a milder profile centered on microenvironmental reorganization rather than transcriptional reprogramming.

BCL2 inhibition also significantly upregulated CCL8 (MCP-2), a chemokine recruiting monocytes, macrophages, and T cells to injured tissue (Supplementary Fig. 3C). Its selective induction in treated, but not Sham or untreated AP groups, likely indicates enhanced apoptotic debris clearance (efferocytosis) and reduced secondary necrosis. Additionally, CCL8-mediated recruitment of phagocytes and anti-inflammatory signaling may further support apoptosis and tissue remodeling [42]. Thus, CCL8 serves both as a marker and a mediator of the shift from necrosis to apoptosis following BCL-2 inhibition in AP.

In the CP model, Navitoclax strongly upregulated ECM components, lysosomal and vesicular proteins while downregulating ribosomal and translation-associated proteins—changes consistent with intensified matrix remodeling and degradation, likely reflecting tissue injury and attempted repair by activated PSCs (Fig. 5E). The suppression of protein synthesis suggests high apoptotic turnover or metabolic stress, aligning with the pronounced fibrosis observed in vivo (Fig. 5F). In contrast, Venetoclax modestly increased ECM- and vesicle-associated proteins but had minimal impact on mitochondrial or translational components, suggesting a less fibrogenic and more tolerable profile in chronic inflammation (Fig. 5G, H).

Notably, both drugs upregulated S100A8 and S100A9 (calgranulins A and B) in AP and CP models (Supplementary Fig. 3D and Fig. 5D). These heterodimers bind Ca^2+^, Zn^2+^, and Mn^2+^, modulating metal ion–dependent signaling and exhibiting antimicrobial activity [43, 44]. Although their short-term upregulation ex vivo may be limited, in vivo these proteins could influence calcium signaling, immune responses, and apoptosis. S100A8/A9 is known to activate phagocyte NADPH oxidase and induce apoptosis [45, 46], contributing to inflammation and cell migration in various diseases [47]. S100A8/A9 complex may also induce cell death through RAGE-independent pathways, influenced by BCL2-family proteins and involving mitochondrial release of OMI/HtrA2 and Smac/DIABLO, XIAP cleavage, and Drp1 downregulation [48]. Interestingly, elevated S100A8/A9 levels predict Venetoclax resistance in acute myeloid leukemia [49], suggesting that modulation of calcium-binding proteins could influence both efficacy and toxicity profiles of BCL2 inhibitors.

The established safety profile of Venetoclax and its clinical use since 2016 for leukemia provide a potential fast-track route for repurposing this drug in AP treatment [7], pending favorable clinical trial outcomes. Our findings offer proof-of-principle and demonstrate the therapeutic potential of an already approved agent, complementing emerging strategies that target Ca^2+^ influx in acinar cells or TRPM2 channel activity [33–35, 50]. This integrated approach not only broadens our understanding of BCL2 inhibitors in gastrointestinal diseases but also highlights their promise in reshaping therapeutic options for AP.

## Materials and methods

### Mouse models of acute and chronic pancreatitis

In vivo experiments (approved by the II Local Ethics Committee in Krakow under approval numbers 226/2018, 106/2020, and 312/2021) were performed on male C57BL6J mice aged 6–8 weeks and weighing 25 ± 4 g, housed in individually ventilated cages at a 12 hour light/dark cycle and with access to water and standard rodent chow. The decision to use only male mice was based on the goal of minimizing biological variability associated with hormonal fluctuations, particularly given the relatively small group sizes. However, the findings should be relevant to both sexes, as the biological pathways and cellular mechanisms targeted in this study are present in both male and female mice. The animals were assigned to experimental groups in a balanced and alternating manner to avoid cage, batch, or handling bias. No formal randomization method was used.

For the EtOH/POA-induced AP model, 30 mice were assigned into six groups: Sham, Navi, Ven, CP, CP Navi, and CP Ven. The AP was induced via two hourly intraperitoneal injections of ethanol (1.5 g/kg) and palmitoleic acid (POA, 300 mg/kg) with PEG200 (1.0 g/kg) (all from Merck, Darmstadt, Germany), following a modified protocol [21]. Venetoclax and Navitoclax (100 mg/kg, from MedChemExpress, Monmouth Junction, NJ, USA), were administered intraperitoneally using a solution of 10% DMSO (Thermo Fisher Scientific, Waltham, MA, USA), PEG200 (Merck), and saline a day before and on the day of induction. Control groups received only the vehicle.

In the cerulein-induced AP model, 36 mice were grouped similarly and subjected to seven hourly intraperitoneal injections of cerulein (50 µg/kg, from MedChemExpress). The treatment groups received Venetoclax/Navitoclax (100 mg/kg) prepared as above, both a day before and on the day of induction, while control groups received the vehicle.

For the CP model, 37 mice were organized into six groups (Sham, Navi, Ven, CP, CP Navi, and CP Ven), receiving cerulein (50 µg/kg) or saline injections seven times at one-hour intervals, twice weekly for eight weeks, totaling 16 injections. From the fifth to the eighth week, Venetoclax or Navitoclax (100 mg/kg) or vehicle was administered twice weekly, a day before cerulein administration, totaling eight administrations.

Animals were euthanized 24 h after AP induction in AP models or 72 h after the last cerulein injection in the CP model by CO_2_ overdose. The pancreata were collected for histological analysis in AP models and split for histology and proteomic analyses (snap-freezing in liquid nitrogen) in the CP model. Blinding was not applied during treatment administration or sample collection.

### Histology

The pancreata and lungs were isolated from the euthanized experimental mice and fixed in 10% formalin for 48 h. The tissues were subsequently processed in ethanol and xylene, embedded in paraffin, and sectioned into 5 μm slices according to a previously published protocol [19]. These sections were stained with hematoxylin and eosin (H&E) as previously described [19] or with Sirius Red. Briefly, for Sirius Red staining, the tissue slices on slides were initially rehydrated (xylene, ethanol 99.8%, 96%, 70%, 50%, dH_2_O), and then the tissues were stained for 1 hour in a solution of 2% picric acid with 0.1% Direct Red 80 (Merck). After staining, the tissues were rinsed twice in 0.5% acetic acid solution. The tissues were successively dehydrated in increasing concentrations of alcohol (70%, 96%, 99.8%), then xylene and the slides were sealed with DPX mountant for histology (Merck). The stained tissues were imaged using a Leica DMi8 microscope (Leica Microsystems, Wetzlar, Germany) with a 20× objective lens (40× for lungs) or via tile-scans of the entire stained sections using a 10× objective.

### Histological scoring

Evaluation was conducted blindly by two independent investigators, one of whom was a physician (AP models). The tissues were scored from 0−27 according to a previously published scale that considered edema (0−3), inflammatory infiltration (0−3), fat necrosis (0/3/5/7), parenchymal necrosis (0/3/5/7), and hemorrhages (0/3/5/7) [20].

For the CP model, a modified histological grading scale was introduced, based on previously published work [20, 25]. The pancreata were assessed on a scale of 0−27, evaluating parenchymal integrity (0/3/5/7), inflammatory infiltration (0−3), acinar atrophy (0/3/5/7), pseudotubular co*mplexe*s (0−3), and fibrosis (0/3/5/7). Fibrosis in the CP model, identified through Sirius Red staining, and the percentage of necrotic areas in the AP model, identified through H&E staining, were quantified using QuPath 0.4.4 software, analyzing whole tissue slice scans [51].

Histological assessment of lung injury in AP models was performed by a physician based on 20 randomly selected fields per sample, acquired from a single hematoxylin- and eosin-stained section using a Leica DMi8 microscope (40× objective). Lung injury was scored on a 0–1 scale (with 0 indicating healthy tissue) according to the criteria described by Matute-Bello et al. [52], evaluating parameters such as neutrophil infiltration, hyaline membrane formation, proteinaceous debris in airspaces, and alveolar septal thickening.

### Immunohistofluorescence

Immunohistofluorescence (IHF) staining for cleaved caspase-3 (Cell Signaling Technology Inc, Danvers, MA, USA; #9661) was conducted according to our standard protocol, incorporating Sudan Black to quench pancreatic autofluorescence [19]. Slides were sealed with ProLong Diamond Antifade mountant with DAPI (Thermo Fisher Scientific). The entire tissue sections were imaged using a tile-scan with a Leica DMi8 automated microscope (20× objective) and representative images were taken with a 40× objective with a ZEISS LSM 880 confocal microscope (Carl Zeiss AG, Oberkochen, Germany) for the EtOH/POA-induced AP model and a Leica DMi8 microscope for the cerulein-induced AP model. Quantitative analysis of the staining results was performed using the QuPath 0.4.4 program [51].

### Pancreatic enzyme measurements and blood analysis

Bood samples from mice in the cerulein-induced and EtOH/POA-induced AP models were collected postmortem from the retro-orbital sinus using K_3_EDTA. In contrast, blood in the CP model was collected at three different time points and into two types of tubes: heparin- and K3EDTA-coated tubes. The collection points were 18 h after the first administration of cerulein (via submandibular route), at week 5 of the experiment on the day therapy commenced (via submandibular route), and postmortem at the end of the experiment during dissection (via retro-orbital sinus). Hematological examinations of the blood were performed using the SCIL ABC VET automatic blood analyzer (Horiba, Kyoto, Japan).

Blood collected in heparin-coated tubes was centrifuged at 2000 g for 10 min for plasma analysis. The plasma was then stored at 4°C for up to 2 days before measurement. The enzymatic activities of amylase and pancreatic lipase were evaluated using colorimetric kits from Cormay (Lomianki, Poland), specifically the Liquick Cor AMYLASE 30 and CORMAY LIPASE kits. These assays were performed on multi-well plates according to the manufacturer’s instructions, with measurements taken using the Synergy H1 Biotek reader (BioTek Instruments, Agilent Technologies, Winooski, VT, USA).

### Isolation of mouse pancreatic acinar cells

For experiments involving pancreatic acinar cells (PACs), the cells were freshly isolated from the mouse pancreas following our standard isolation procedure [16]. Male C57BL6J mice (6–8 weeks old, 25 ± 4 g) were euthanized by cervical dislocation. The pancreas was then washed twice in NaHEPES buffer (140 mM NaCl, 4.7 mM KCl, 10 mM HEPES, 1 mM MgCl_2_, 10 mM glucose, and 1.0 mM CaCl_2_, with a pH of 7.2) [53]. HEPES sodium salt and other salts for the preparation of NaHEPES buffer were purchased from VWR (Radnor, PA, USA). The pancreas was repeatedly injected with approximately 400−600 µL of collagenase IV solution 0.75 mg/mL (Merck) and incubated for 7−9 min at 37°C. Using a clipped automatic pipette tip, the pancreatic tissue was mechanically fragmented by repeated pipetting. The supernatant containing PACs was collected, centrifuged twice at 200 g for 2 min, and finally resuspended in NaHEPES buffer. The isolated cells were used within a maximum of 4 h after isolation and stored at 4°C until use.

### In vitro model of hPSC cells activation

Human pancreatic stellate cells (hPSC, #3830) and culture medium (SteCM, #5301) with 2% FBS, CGS supplement and P/S were obtained from ScienCell (Carlsbad, CA, USA). Quiescent (qhPSC) cells were cultured in complete SteCM in standard conditions previously described [19]. Spontaneous activation of cells (shPSCs) was carried out in incomplete medium (lacking FBS and CGS) for 48 h (shPSC cells). Activated cells (ahPSCs) were cultured in incomplete medium supplemented with 5 ng/mL TGF-β (Corning, New York, NY, USA) for 48 h. To ensure the cells were free of mycoplasma, regular PCR assays were performed.

### RNA Isolation and RT-qPCR

Human pancreatic stellate cells (hPSCs) were cultured in 6-well plates. Total RNA was isolated using Fenozol Plus and the Total RNA Mini Kit (A&A Biotechnology, Gdańsk, Poland) according to the manufacturer’s instructions. 1 µg of total RNA was used for cDNA synthesis with the High Capacity cDNA Reverse Transcription Kit (Thermo Fisher Scientific), following the manufacturer’s protocol. RT-qPCR was performed using the QuantStudio™ 12 K Flex Real-Time PCR System under the following conditions: 50°C for 20 seconds and 95°C for 5 min, followed by 40 cycles of 95 °C for 15 seconds and 60°C for 1 minute. Reactions were carried out using the GoTaq® qPCR Master Mix (Promega, Madison, WI, USA) in a MicroAmp™ Fast Optical 96-Well Reaction Plate. Relative gene expression levels were determined using the comparative Ct (2^–ΔΔCt) method, with *RPL28* (60S ribosomal protein L28) used as the reference gene. The sequences of primers (Genomed, Warsaw, Poland) were as follows: for *ACTA2* (α-SMA), forward primer ACTGCCTTGGTGTGTGACAA and reverse primer CACCATCACCCCCTGATGTC; for *RPL28*, forward primer GACCTACAGCACTGAGCCCAATAAC and reverse primer TGGTGGTCCGCACATAGGA.

### Cell death measurements

To assess mouse PAC death, isolated cells in NaHEPES buffer were pre-treated with Venetoclax or Navitoclax at a concentration of 10 µM, or with the appropriate amount of DMSO as a control, for 15 min. Following this pre-treatment, the cells were stimulated for 1 hour with either 10 nM cerulein or a combination of 200 mM ethanol and 200 µM palmitoleic acid (EtOH/POA). Simultaneously, one hour before imaging, the cells were stained using the NucView 488 & RedDot 2 Apoptosis and Necrosis Kit (Biotium, Fremont, CA, USA) following the manufacturer’s protocol. This kit allows for the visualization of caspase 3 and 7 activity in green fluorescence and necrosis in far red fluorescence. PACs were then transferred to a glass-bottom chamber and imaged using a DMI8 fluorescence microscope equipped with an HC PL APO 40× / 1.30 OIL objective and a DFC7000GT camera (all from Leica). The imaging parameters for NucView 488 were as follows: excitation at 490 nm, 5% illumination power, and an FITC emission filter. For RedDot 2 imaging, the parameters were: excitation at 660 nm, 9% illumination power, and a CY5 emission filter. A total of 15 random images were collected for each condition. The total number of cells, apoptotic cells, and necrotic cells were counted manually.

To assess hPSC death, cells were seeded in a 24-well plate. Cells were then stimulated with Navitoclax or Venetoclax or DMSO (control) at a concentration of 100 nM for 48 h with simultaneous activation (spontaneous shPSCs or with TGF-β, ahPSCs) or without activation (qhPSCs). Hoechst 33342 (Thermo Fisher Scientific) at a concentration of 1 ug/mL and Red Dot 2 (Biotium) diluted according to the manufacturer’s instructions were added to the cells for 30 min before measurement without changing or washing off the medium. A series of images of cells were taken then under a Leica DMi8 microscope, equipped with an N PLAN L 20×/0.35 DRY objective and a DFC7000GT camera (all from Leica). The imaging parameters for Hoechst 33342 were as follows: excitation at 365 nm, 5% illumination power, and an DAPI emission filter. For RedDot 2 imaging, the parameters were: excitation at 660 nm, 13% illumination power, and a CY5 emission filter. From each condition tested, 4 random images were taken from 2 wells. The percentage of dead cells was then calculated using QuPath 0.4.4 program. The percentage of cell death was calculated for each individual experiment, averaged, and presented as mean ± SD.

### ATP measurements

The CellTiter-Glo 3D Cell Viability Assay (Promega) was used to measure the amount of ATP produced by isolated pancreatic acinar cells. As with the previous setup, cells were pre-incubated with either Venetoclax or Navitoclax at 10 µM, or DMSO as a control, and then stimulated with 10 nM cerulein or a 200 mM / 200 µM EtOH/POA solution in NaHEPES buffer. After stimulation, the cells were transferred to a 96-well white/clear bottom plate, an equal volume of the CellTiter-Glo reagent was added, the plate was shaken for 5 min, and then left for 25 min at room temperature to stabilize the samples. The luminescence signal was read using a Synergy H1 BioTek reader. The results were normalized to the total amount of protein in each sample, using the same volume of cell suspension for the measurement, after performing a BCA protein assay.

### Calcium signaling

Isolated PACs were loaded with Fluo-4 AM dye (Thermo Fisher Scientific) at a concentration of 5 µM in NaHEPES buffer for 30 min at room temperature. After loading, the cells were washed in NaHEPES, centrifuged at 200 g for 2 min, and then stored at 4°C until the experiment. For experiments conducted without Ca^2+^ ions, NaHEPES buffer without CaCl_2_ was used. In some experiments, cells were pre-stimulated with Venetoclax or Navitoclax at a concentration of 10 µM for 15 min before imaging. PACs were transferred to a glass-bottom perfusion chamber, connected to a continuous aspiration / perfusion system with NaHEPES-based solutions. For calcium signaling measurements in hPSCs, cells were seeded onto 32 mm glass coverslips placed in plastic Petri dishes and either left quiescent (qhPSCs) or activated (ahPSCs). Prior to imaging, cells were loaded with 2 µM Fluo-4 AM in NaHEPES buffer for 30 min. Calcium signaling was then measured using the same protocol as for PACs.

Images were acquired using a DMI8 fluorescence microscope equipped with an HC PL APO 40 × /1.30 OIL objective and a DFC7000GT camera (all from Leica). The imaging parameters included image acquisition every 1 or 2 seconds, depending on the experiment, with 2% illumination power of the LED light source at 490 nm, an FITC emission filter, and 5 × 5 binning (resolution: 384 × 288 pixels). Fluorescence signals were plotted as F/F_0_, where F_0_ was the averaged signal from the first ten baseline images of one region of interest (ROI), normalized as previously described [18, 54]. Based on these data, the area under the curve and the maximum amplitude of the response were calculated.

### Sample preparation and LC-MS/MS for CP model

Mouse pancreata from the CP model were lysed using a TissueLyser homogenizer (Qiagen, Hilden, Germany) in a buffer consisting of 0.1 M Tris (pH 7.5) and 1% SDS, adjusted according to the tissue size. Then the tissue lysates were subjected to sonication for 7 min using a 30 second ON / 30 second OFF cycle in a Bioruptor Pico (Diagenode, Seraing, Belgium). Following sonication, the lysates were centrifuged, and the supernatants were collected. Proteins (80 µg) were prepared for LC-MS/MS analysis employing the filter-aided sample preparation (FASP) method [55]. Briefly, proteins were reduced with 50 mM dithiothreitol for 15 min at room temperature (RT), then transferred into filtration columns VIVACON 500 (Sartorius Stedim Biotech GmbH, Aubagne, France). They were washed with an 8 M urea solution in 50 mM ammonium bicarbonate and subsequently alkylated with 54 mM iodoacetamide for 20 min in the dark at RT. Following this, samples were washed three times with the urea solution and then three times with 50 mM ammonium bicarbonate. Trypsin was added to the columns for overnight protein digestion at 37°C. The resulting peptides were collected by centrifugation, with further washes using 50 mM ammonium bicarbonate and 0.5 M NaCl. The peptides were then vacuum dried and resuspended in a loading buffer (2% acetonitrile with 0.05% trifluoroacetic acid). Approximately 1 µg of peptides was injected into a nanoHPLC (UltiMate 3000 RSLCnano System, Thermo Fisher Scientific). Initially, samples were concentrated and desalted on a C18 pre-column (Acclaim Pep-Map 100, Thermo Fisher Scientific; ID 75 µm, length 20 mm, particle size 3 µm, pore size 100 Å) and then separated on a 50 cm C18 analytical column (Acclaim PepMap RSLC, Thermo Fisher Scientific; ID 75 µm, particle size 2 µm, pore size 100 Å) using a 4 hour gradient of acetonitrile (2%–40%) in the presence of 0.05% formic acid at a flow rate of 250 nL/min. Eluting peptides were ionized using a Digital PicoView 550 nanospray source (New Objective, Littleton, MA, USA) and analyzed by a Q Exactive mass spectrometer (Thermo Fisher Scientific) in data-dependent mode using the Top12 method. Full MS and MS/MS spectra were acquired at resolutions of 70,000 and 17,500, respectively. The performance of the LC-MS/MS system was monitored using the QCloud quality control system [56].

### LC-MS/MS data analysis for CP model

The acquired data were processed using MaxQuant software (version 2.1.4.0) [57], and searched with the integrated Andromeda search engine against the UniProtKB database [58], which was restricted to the *Mus musculus* taxonomy (17,148 sequences; downloaded March 7, 2023). The false discovery rate (FDR) for peptide and protein identification was set at 1%. Label-free quantification (LFQ) was performed. The resulting MaxQuant output table, which included protein group identifications and quantitative information (LFQ intensities), was uploaded to the Perseus platform (version 2.0.10.0) for further processing [59].

Initially, the list of identified protein groups was refined by excluding proteins identified in the decoy database, contaminants, and proteins only identified by site. Subsequently, LFQ intensities were log2 transformed, and the data matrix was filtered to include proteins with at least four valid values in one or more groups. To detect significant differences between experimental groups, a Student’s *t*-test was performed, followed by permutation-based FDR correction set at 5%. A minimum of four valid LFQ intensity values in at least one of the two tested groups was required to conduct the test for each comparison. Only proteins identified based on at least two peptides were included in the final sets of differential proteins obtained for subsequent comparisons.

### Sample preparation and LC-MS/MS for cerulein AP model

Samples were prepared as previously described for the CP model. Protein samples (20 µg) were processed for LC-MS/MS analysis using the SP3 sample preparation protocol [60]. The resulting peptides were resuspended in 0.1% formic acid (FA), and 250 ng of the peptide mixture was analyzed using an Astral Orbitrap mass spectrometer coupled to a Vanquish Neo UHPLC system (both Thermo Fisher Scientific). The UHPLC was operated in direct injection mode. Peptides were separated on an Aurora Ultimate XT 25 cm × 75 µm C18 column (IonOpticks, Fitzroy, Australia) using a 60 minute acetonitrile (ACN) gradient at a flow rate of 0.5 µL/min and a column temperature of 55 °C. The gradient consisted of three steps: 2–10% buffer B over 10 min, 10–25% B over 32 min, and 25–45% B over 18 min (buffer B: 80% ACN with 0.1% FA; buffer A: 0.1% FA).

Eluting peptides were analyzed on the Orbitrap Astral mass spectrometer using a data-independent acquisition (DIA) method. Data were acquired within 200 windows of 3 Th across the precursor mass range of 380–980 m/z. The maximum injection time (IT) was set to millisecond, with a normalized AGC target of 500%. Isolated ions were fragmented using a normalized collision energy of 25%. The Astral scan range was set to 150–2000 m/z. In addition, full-MS spectra were acquired every 0.6 second with a resolution of 240,000, a scan range of 380–980 m/z, a maximum IT of 3 millisecond, and a normalized AGC target of 500%.

### LC-MS/MS data analysis for AP model

Raw files were analyzed using DIA-NN version 2.0.2 [61], employing an in silico predicted spectral library generated from the mouse reference proteome (UniProtKB; 21,755 sequences; downloaded in February 2025). Both spectral library generation and peptide/protein identification were performed using the following DIA-NN settings: protease specificity set to Trypsin/P with up to one missed cleavage allowed; maximum number of variable modifications set to two, including carbamidomethylation of cysteine, methionine oxidation, N-terminal methionine excision, and protein N-terminal acetylation. The peptide length range was set to 7–30 amino acids, with a precursor charge range of 2–4, precursor m/z range of 380–980, and fragment ion m/z range of 150–2000. Mass accuracy was set to 10 ppm for fragments and 4 ppm for precursors. Scan window size was 7, and scoring was based on peptidoforms, with proteotypicity assigned at the gene level. Neural networks (cross-validated) were used for machine learning, and quantification was performed using the QuantUMS (high-precision) strategy. Cross-run normalization was RT-dependent, and library generation included ID-based retention time (RT) and ion mobility (IM) profiling. Speed and RAM usage were optimized for performance. Match-between-runs (MBR) and protein inference were enabled.

The resulting pg_matrix output was filtered to include only protein groups meeting the Global.PG.Q.Value threshold of 0.01. Filtering was conducted in RStudio (v2024.12.1 + 563), followed by removal of common repository of adventitious proteins (cRAP). The data matrix was then processed in Perseus (v2.0.11.0) [59]. Protein intensity values were log₂-transformed, and proteins with at least five valid values in at least one group were retained. Remaining missing values were imputed using a constant low-intensity value. Differential protein abundance was assessed using Student’s *t*-test with permutation-based FDR set at 5%. Proteins with a *q*-value < 0.05 and an abundance change of ≥20% were considered differentially expressed.

### Functional analysis of differential proteins

For the functional analysis, lists of differential proteins were further refined by considering a minimum fold change threshold. Proteins with at least a 1.2-fold change were selected for the Navi versus Sham and CP/Ven versus CP comparisons. For the CP versus Sham and CP/Navi versus CP comparisons, a fold change threshold of 2 was applied. Proteins showing at least a 1.2-fold change were selected for comparisons in the cerulein-induced AP model. Gene Ontology (GO) enrichment analysis for Cellular Component was performed using ShinyGO v0.82 [62].

### Statistical analysis and language editing

Statistical analyses were conducted using GraphPad software. Sample sizes for cell-based experiments were not predetermined using statistical methods. For animal studies, the number of subjects in each group was determined based on the guidelines from Charan and Kantharia, 2013 [63]. Inclusion criteria encompassed all animals and samples prepared according to the study protocol. No pre-established exclusion criteria were applied, and no data points were removed from analysis post hoc. The normality of data distributions was tested using the Shapiro-Wilk test. For datasets normally distributed either one-way ANOVA followed by Sidak’s multiple comparisons test or unpaired *t*-tests with Welch’s correction were applied. Equality of variance was evaluated based on descriptive statistics and visual assessment of group distributions. For data failing the normality test, a non-parametric Kruskal-Wallis test followed by Dunn’s post hoc test was used. A significance level was established at a *p*-value of 0.05, with adjustments made for multiple comparisons where necessary. Each figure legend specifies the statistical tests employed. Unless otherwise stated ‘N’ refers to the number of independent experimental repetitions, while ‘n’ represents the count of individual cells. Parts of the text were corrected using AI academic writing tools and large language model tools.

## Supplementary information

Supplementary Information

## Data Availability

Raw data from individual experiments are available upon reasonable request. The mass spectrometry data were deposited to the ProteomeXchange Consortium via the MassIVE repository with the dataset identifier PXD055578 and via the PRIDE partner repository with the dataset identifier PXD064881 [64, 65].
