# Supplementary material for: Repurposing BCL2 inhibitors: Venetoclax protects against acinar cell necrosis in acute pancreatitis by promoting apoptosis
Source: Cell Death Dis. 2025 Jul 27;16(1):566. doi: 10.1038/s41419-025-07881-w (PMC12297356; doi:10.1038/s41419-025-07881-w)

SUPPLEMENTARY INFORMATION

Litewka et al., 2025, Cell Death & Disease

Supplementary Figure Legends

Supplementary Figure 1: Navitoclax and Venetoclax in cerulein and EtOH/POA-induced models of AP – pulmonary histology

A: Representative hematoxylin and eosin (H&E) stained images of mouse lungs from the cerulein-induced AP model. Scale bar: 50 µm. Images taken with a 40× magnification objective. The black arrowhead indicates thickening of the interalveolar septa accompanied by an inflammatory neutrophilic infiltrate; the white arrowhead indicates the presence of neutrophils within the alveolar lumen; the asterisk denotes hyaline membranes and proteinaceous material within the alveolar lumen; and the plus sign marks the normal appearance of alveoli.

B: Overall histological pulmonary score (0-1) for each group in the cerulein-induced AP model [1]. Results are presented as mean ± SD.

C: Representative hematoxylin and eosin (H&E) stained images of mouse lungs from the EtOH/POA-induced AP model. Scale bar: 50 µm. Images taken with a 40× magnification objective. The black arrowhead indicates thickening of the interalveolar septa accompanied by an inflammatory neutrophilic infiltrate; the white arrowhead indicates the presence of neutrophils within the alveolar lumen; the asterisk denotes hyaline membranes and proteinaceous material within the alveolar lumen; and the plus sign marks the normal appearance of alveoli.

D: Overall histological pulmonary score (0-1) for each group in the EtOH/POA-induced AP model [1]. Results are presented as mean ± SD.

Statistical Analyses: For the cerulein-induced AP model, n=6 for all groups; for the EtOH/POA-induced AP model, n=5, except for the AP Navi group with n=4. Data normality was assessed using the Shapiro-Wilk test. Since the data followed a normal distribution, statistical analyses were performed using ordinary one-way ANOVA.

Figure Abbreviation Legend: Sham (saline + vehicle), Navi (saline + Navitoclax), Ven (saline + Venetoclax), AP (cerulein or EtOH/POA + vehicle), AP + Navi (cerulein or EtOH/POA + Navitoclax), AP + Ven (cerulein or EtOH/POA + Venetoclax).

Supplementary Figure 2: Calcium signaling in PACs treated with BCL2 inhibitors

A: Representative traces of intracellular Ca^{2+} responses recorded in qhPSCs upon acute treatment with Navitoclax, Venetoclax (10 µM), or no treatment (control) for 10 minutes (between 200-800 seconds of the recording), followed by application of high concentrations of acetylcholine (ACh 10 µM) for 200 seconds (between 800-1000 seconds of the experiment). Data for the representative traces and subsequent analyses were obtained from qhPSCs loaded with Fluo-4 AM dye, in a continuous perfusion flow system using NaHEPES buffer, from six independent experiments for each group (N=6, n=90 cells). For all conditions, cells were derived from 3 independent passages, with each contributing to 2 experiments, 15 cells per experiment.

B: Normalized area under the Ca^{2+} signaling traces from 200-800 seconds in qhPSCs after acute treatment with Navitoclax, Venetoclax (10 µM), or no treatment (control); representative traces are depicted in A. Results are presented as mean ± SD.

C: Normalized maximal amplitude from Ca^{2+} signaling traces between 200-800 seconds in qhPSCs following acute application of Navitoclax, Venetoclax (10 µM), or no treatment (control); representative traces are depicted in A. Results are presented as mean ± SD.

D: Normalized area under the Ca^{2+} signaling traces from 800-1000 seconds in qhPSCs during application of high concentration of ACh (10 µM) after pretreatment with Navitoclax,

Venetoclax (10 μM), or no pretreatment (control); representative traces are depicted in A. Results are presented as mean ± SD.

E: Normalized maximal amplitude from Ca^{2+} signaling traces between 800-1000 seconds in qhPSCs during application of high concentration of ACh (10 μM) after pretreatment with Navitoclax, Venetoclax (10 μM), or no pretreatment (control); representative traces are depicted in A. Results are presented as mean ± SD.

F: Representative traces of intracellular Ca^{2+} responses recorded in ahPSCs upon acute treatment with Navitoclax, Venetoclax (10 μM), or no treatment (control) for 10 minutes (between 200-800 seconds of the recording), followed by application of high concentrations of acetylcholine (ACh 10 μM) for 200 seconds (between 800-1000 seconds of the experiment). Data for the representative traces and subsequent analyses were obtained from ahPSCs loaded with Fluo-4 AM dye, in a continuous perfusion flow system using NaHEPES buffer, from six independent experiments for each group (N=6, n/t: n=80 cells, Venetoclax: n=75 cells, Navitoclax: n=85 cells). For all conditions, cells were derived from 3 independent passages, with each contributing to 2 experiments, 10-15 cells per experiment.

G: Normalized area under the Ca^{2+} signaling traces from 200-800 seconds in ahPSCs after acute treatment with Navitoclax, Venetoclax (10 μM), or no treatment (control); representative traces are depicted in F. Results are presented as mean ± SD.

H: Normalized maximal amplitude from Ca^{2+} signaling traces between 200-800 seconds in ahPSCs following acute application of Navitoclax, Venetoclax (10 μM), or no treatment (control); representative traces are depicted in F. Results are presented as mean ± SD.

I: Normalized area under the Ca^{2+} signaling traces from 800-1000 seconds in ahPSCs during application of high concentration of ACh (10 μM) after pretreatment with Navitoclax, Venetoclax (10 μM), or no pretreatment (control); representative traces are depicted in F. Results are presented as mean ± SD.

J: Normalized maximal amplitude from Ca^{2+} signaling traces between 800-1000 seconds in ahPSCs during application of high concentration of ACh (10 μM) after pretreatment with Navitoclax, Venetoclax (10 μM), or no pretreatment (control); representative traces are depicted in F. Results are presented as mean ± SD.

Statistical analyses: Data distribution was assessed for normality using the Shapiro-Wilk test, which confirmed that the data were not normally distributed. Consequently, non-parametric statistical analyses were performed using the Kruskal-Wallis test followed by Dunn's post hoc test to assess differences between groups.

Figure abbreviation legend: n/t (non-treatment, that is in NaHEPES buffer only), Navi (Navitoclax 10 μM), Ven (Venetoclax 10 μM), Cer (cerulein 10 nM), ACh 10 μM (acetylcholine 10 μM).

Supplementary Figure 3. Proteomic analysis in the cerulein-induced AP model

A: Principal component analysis (PCA) of all samples based on their proteomic profiles. Different experimental groups are displayed in various colors (n=6 for all groups).

B: Percentage of differentially expressed proteins identified based on at least two peptides. Comparisons were performed using Student's t-test followed by permutation-based false discovery rate (FDR) correction (q-value < 0.05). Each protein included in the analysis was required to have at least four valid label-free quantification (LFQ) intensity values in at least one of the two compared groups.

C: Average levels of CCL8 (C-C motif chemokine 8) across different experimental groups. Mean values were calculated from log2-transformed LFQ intensities. CCL8 was not quantified in the Sham and AP groups. In the Ven group, CCL8 was quantified in 3 samples, and in the Navi group, in 4 samples. Missing values, predominantly resulting from very low protein abundance below the detection limit of the LC-MS/MS system, were imputed with an arbitrary

low value (8.4 log2 LFQ intensity) prior to statistical analysis. Results are presented as means. Statistical analysis was performed using Student's t-test. ***$q < 0.001$.

D: Average levels of S100A8 and S100A9 proteins across different experimental groups. Mean values were calculated from log2-transformed LFQ intensities. Missing values, mainly reflecting protein levels below the detection threshold, were imputed prior to analysis. Results are presented as means. Statistical analysis was performed using Student's t-test. **$q < 0.01$, marked statistical significance in the graph applies to both proteins tested.

E: Gene Ontology (GO) analysis of Cellular Component enrichment for proteins upregulated with a fold change of at least 1.2 in the Navi vs. Sham comparison. Analysis was performed using ShinyGO v0.82.

F: Gene Ontology (GO) analysis of Cellular Component enrichment for proteins upregulated with a fold change of at least 1.2 in the Ven vs. Sham comparison. Analysis was performed using ShinyGO v0.82.

Analyses in E-F were performed using ShinyGO v0.82 [2].

Supplementary Figure 4. Proteomic changes in pancreatic tissues from the cerulein-induced AP model and the CP model compared to Sham controls

A: Gene Ontology (GO) analysis of Cellular Component enrichment for proteins upregulated with a fold change of at least 1.2 in the AP vs. Sham comparison. Analysis was performed using ShinyGO v0.82.

B: Gene Ontology (GO) analysis of Cellular Component enrichment for proteins downregulated with a fold change of at least 1.2 in the AP vs. Sham comparison. Analysis was performed using ShinyGO v0.82.

C: Gene Ontology (GO) analysis of Cellular Component enrichment for proteins upregulated with a fold change of at least 2 in the CP vs. Sham comparison. Analysis was performed using ShinyGO v0.82.

D: Gene Ontology (GO) analysis of Cellular Component enrichment for proteins downregulated with a fold change of at least 2 in the CP vs. Sham comparison. Analysis was performed using ShinyGO v0.82.

All analyses were performed using ShinyGO v0.82 [2].

Supplementary References

1. Matute-Bello G, Downey G, Moore BB, Groshong SD, Matthay MA, Slutsky AS, et al. An official American Thoracic Society workshop report: features and measurements of experimental acute lung injury in animals. Am J Respir Cell Mol Biol. 2011;44:725-38.
2. Ge SX, Jung D, Yao R. ShinyGO: a graphical gene-set enrichment tool for animals and plants. Bioinformatics. 2020;36:2628-9.

Supplementary Figure 1

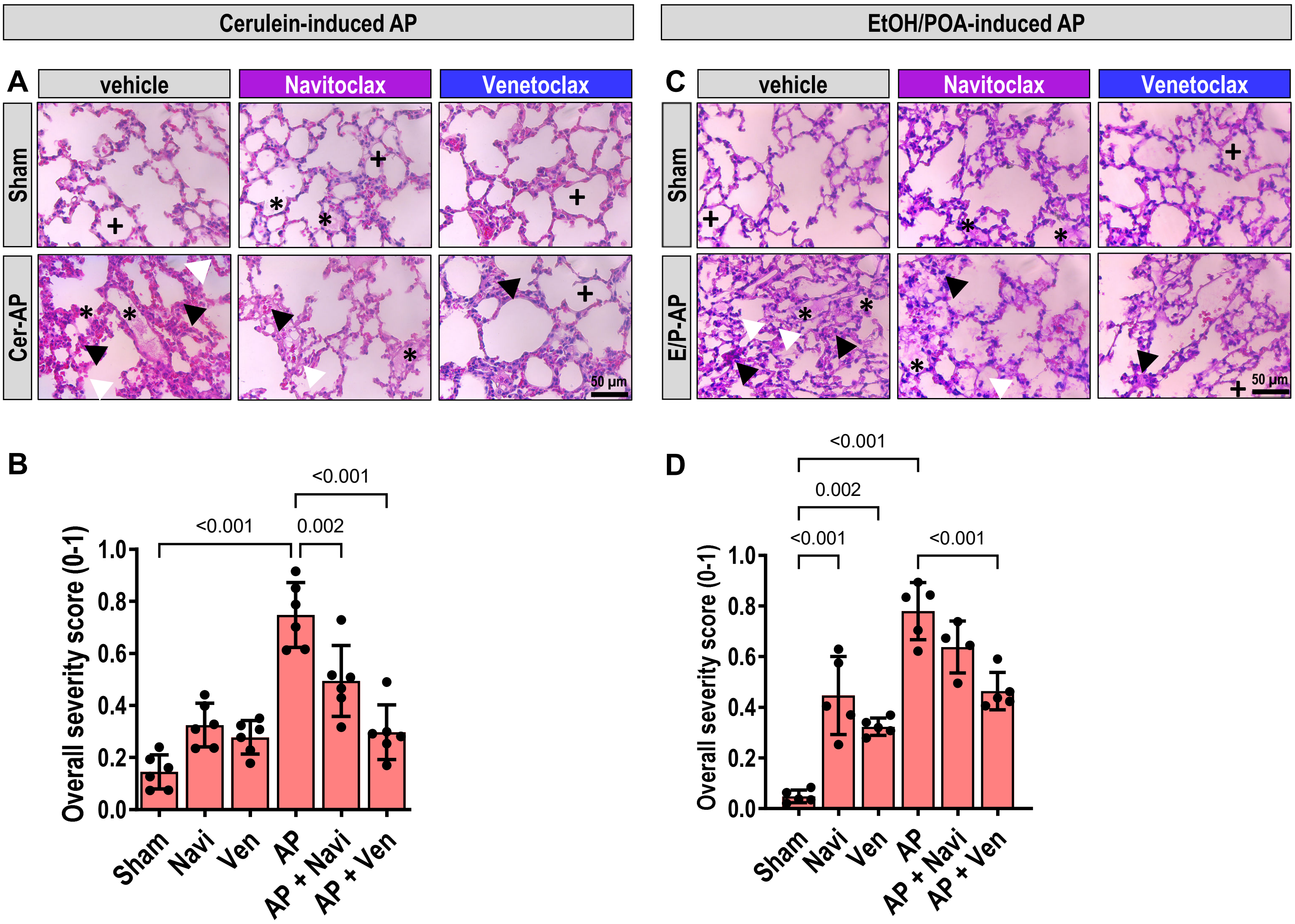

Supplementary Figure 2

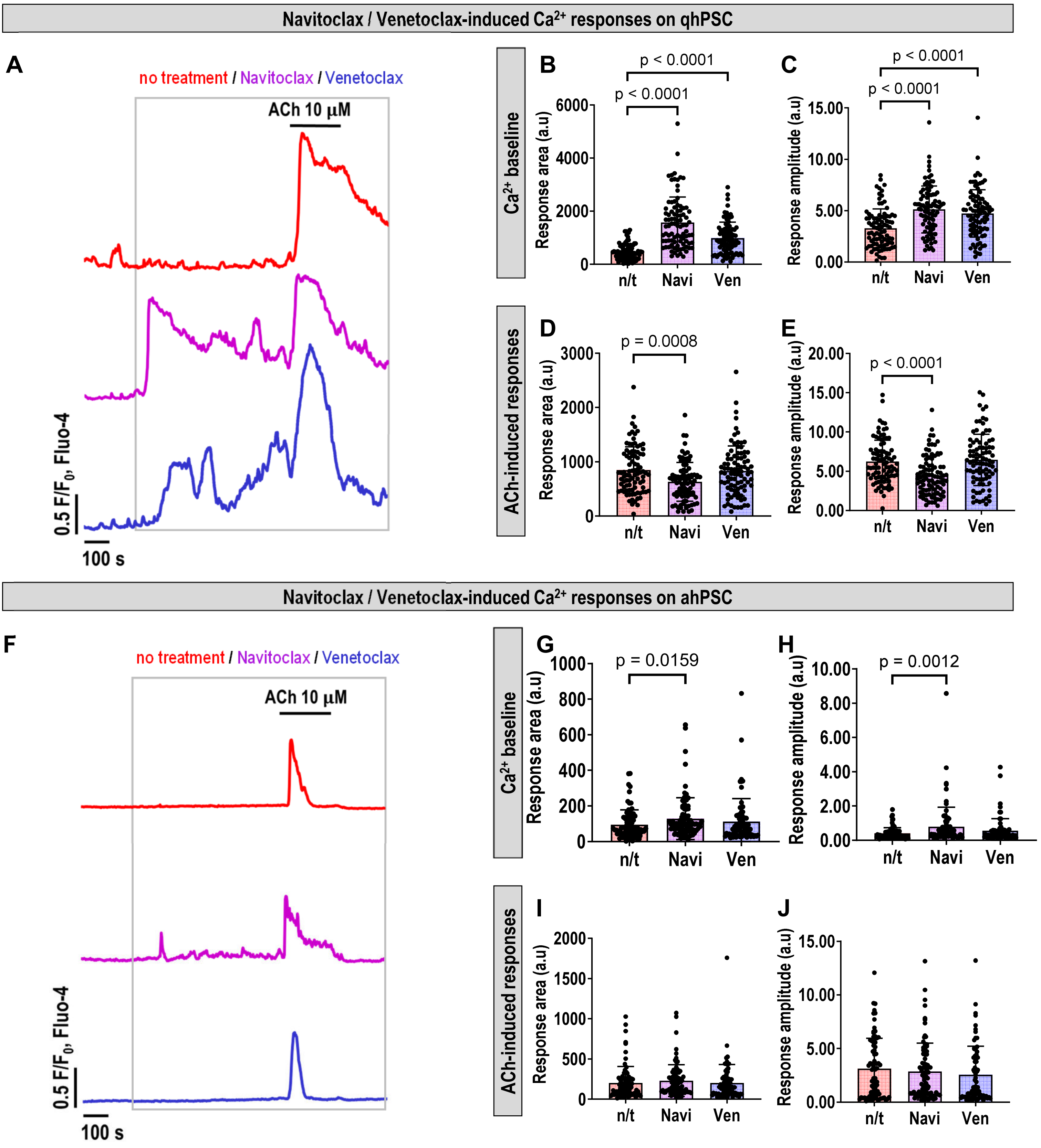

Supplementary Figure 3

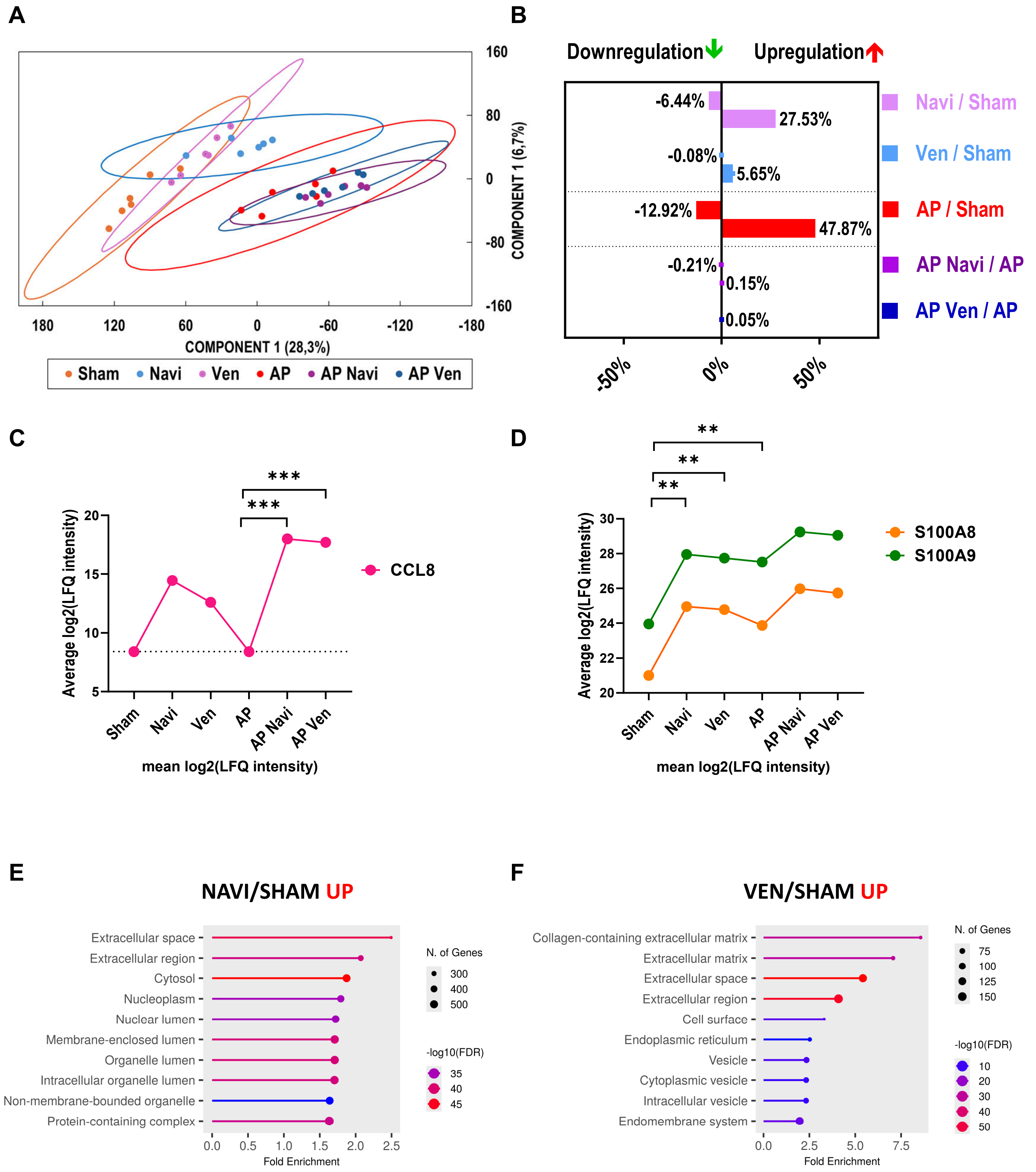

Supplementary Figure 4

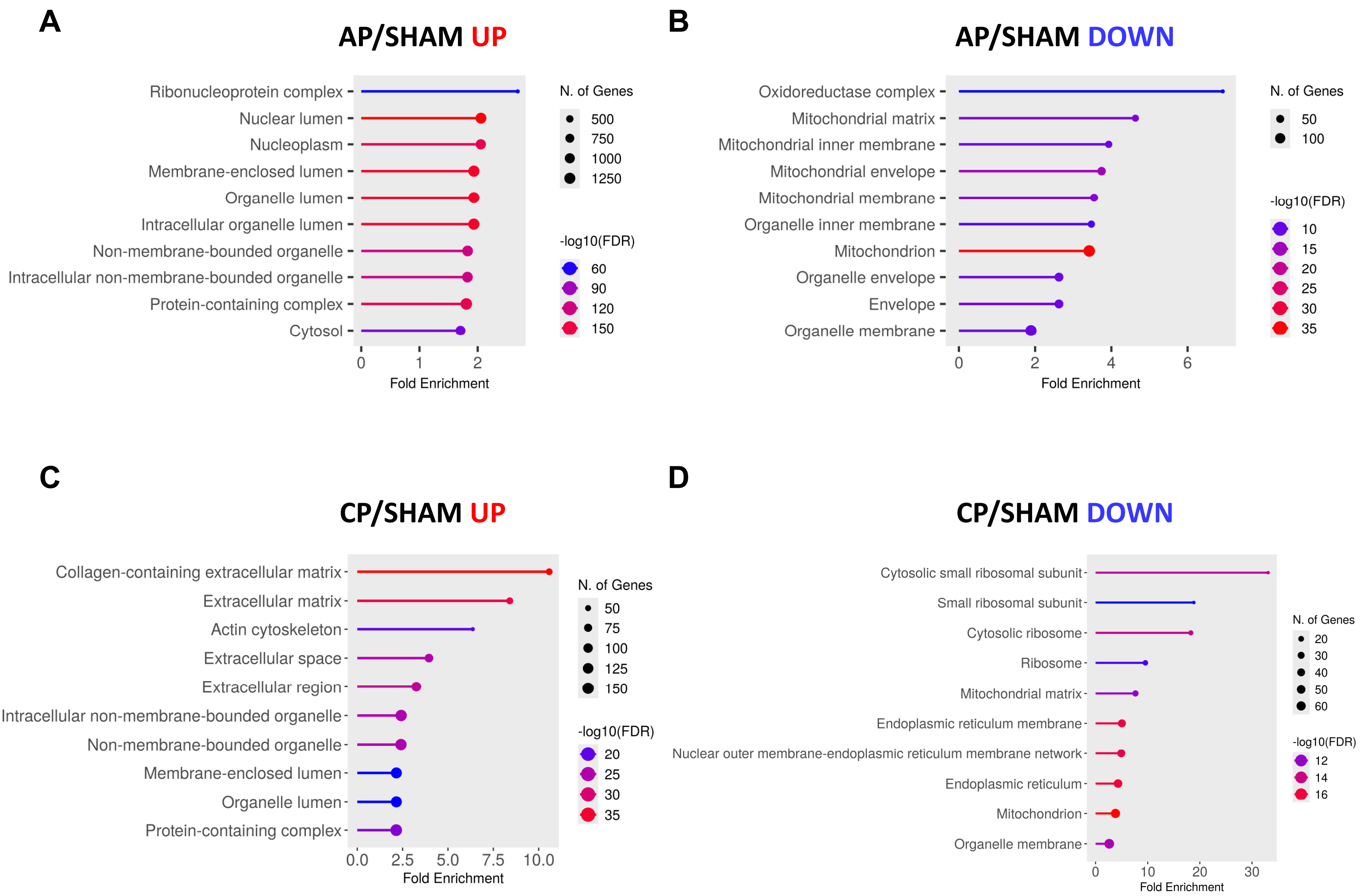

Supplement: Supplementary file 1 — Supplementary Information [file 41419_2025_7881_MOESM1_ESM.pdf]
